# The role of microRNA-155/liver X receptor pathway in experimental and idiopathic pulmonary fibrosis

**DOI:** 10.1016/j.jaci.2016.09.021

**Published:** 2017-06

**Authors:** Mariola Kurowska-Stolarska, Manhl K. Hasoo, David J. Welsh, Lynn Stewart, Donna McIntyre, Brian E. Morton, Steven Johnstone, Ashley M. Miller, Darren L. Asquith, Neal L. Millar, Ann B. Millar, Carol A. Feghali-Bostwick, Nikhil Hirani, Peter J. Crick, Yuqin Wang, William J. Griffiths, Iain B. McInnes, Charles McSharry

**Affiliations:** a Institute of Infection, Immunity and Inflammation, University of Glasgow, Glasgow, United Kingdom; b Scottish Pulmonary Vascular Unit, University of Glasgow, Glasgow, United Kingdom; c Institute of Cardiovascular and Medical Sciences, University of Glasgow, Glasgow, United Kingdom; d Academic Respiratory Unit, Learning and Research, University of Bristol, Bristol, United Kingdom; e Division of Rheumatology & Immunology, Medical University of South Carolina, Charleston, SC; f University of Edinburgh/MRC Centre for Inflammation Research, the Queen's Medical Research Institute, Edinburgh, United Kingdom; g College of Medicine, Swansea University, Swansea, United Kingdom; h Greater Glasgow and Clyde Clinical Research and Development, Yorkhill Hospital, Glasgow, United Kingdom

**Keywords:** MicroRNA-155, lung fibrosis, liver X receptor, fibroblasts, alternatively activated macrophages, Arg2, Arginase 2, Col, Collagen, IPF, Idiopathic pulmonary fibrosis, LXRα, Liver X receptor alpha, miR-155, MicroRNA-155, 3′UTR, 3-Prime untranslated region, 22(S)HC, 22(S)-hydroxycholesterol, WT, Wild-type

## Abstract

**Background:**

Idiopathic pulmonary fibrosis (IPF) is progressive and rapidly fatal. Improved understanding of pathogenesis is required to prosper novel therapeutics. Epigenetic changes contribute to IPF; therefore, microRNAs may reveal novel pathogenic pathways.

**Objectives:**

We sought to determine the regulatory role of microRNA (miR)-155 in the profibrotic function of murine lung macrophages and fibroblasts, IPF lung fibroblasts, and its contribution to experimental pulmonary fibrosis.

**Methods:**

Bleomycin-induced lung fibrosis in wild-type and miR-155^−/−^ mice was analyzed by histology, collagen, and profibrotic gene expression. Mechanisms were identified by *in silico* and molecular approaches and validated in mouse lung fibroblasts and macrophages, and in IPF lung fibroblasts, using loss-and-gain of function assays, and *in vivo* using specific inhibitors.

**Results:**

miR-155^−/−^ mice developed exacerbated lung fibrosis, increased collagen deposition, collagen 1 and 3 mRNA expression, TGF-β production, and activation of alternatively activated macrophages, contributed by deregulation of the miR-155 target gene the liver X receptor (LXR)α in lung fibroblasts and macrophages. Inhibition of LXRα in experimental lung fibrosis and in IPF lung fibroblasts reduced the exacerbated fibrotic response. Similarly, enforced expression of miR-155 reduced the profibrotic phenotype of IPF and miR-155^−/−^ fibroblasts.

**Conclusions:**

We describe herein a molecular pathway comprising miR-155 and its epigenetic LXRα target that when deregulated enables pathogenic pulmonary fibrosis. Manipulation of the miR-155/LXR pathway may have therapeutic potential for IPF.

## Graphical abstract

**Figure:**
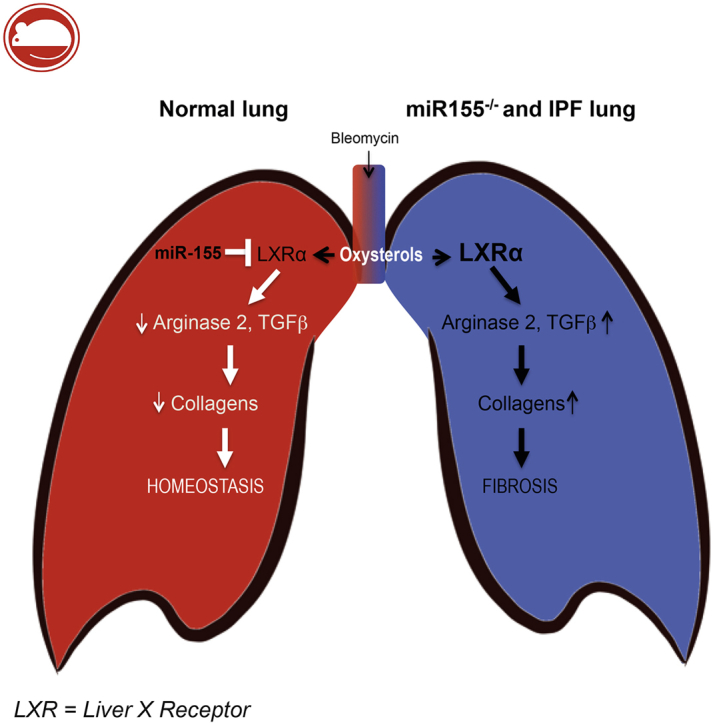

Idiopathic pulmonary fibrosis (IPF) affects more than 5 million people worldwide and its incidence is increasing.^1^ Histology of IPF includes interstitial fibroblastic foci and deposition of collagen-rich extracellular matrix,^2^ and pirfenidone-targeting tissue remodeling has improved therapeutic options.^3^ However, mechanisms controlling IPF progression remain poorly understood. IPF is associated with age, male sex, and cigarette smoking,^4^ suggesting an epigenetic contribution to pathogenesis.

MicroRNAs (miRs) are 22-nucleotide noncoding RNAs that regulate gene expression.^5^ Single miRs bind 6 to 8 nucleotide complementary sequences, mainly in the 3-prime untranslated region (3′UTR) of target mRNAs, causing degradation or translation inhibition^6^ and can finetune diverse mRNA often within the same biological pathway.^7^ Identifying disease-specific miRs can reveal novel target mRNA/pathways and provide insight into pathogenesis and identify therapeutic targets.

MicroRNA-155 (miR-155) is required for normal immune function^8^, ^9^; its overexpression is associated with inflammation, autoimmunity,^8^, ^10^ and cancer,^11^ whereas miR-155–deficient mice develop age-related airway fibrosis.^12^ miR-155 may therefore act as a homeostatic rheostat contributing to the onset and duration of inflammation and remodeling. Our hypothesis was that miR-155 attenuates pathways that induce lung remodeling. We revealed exacerbated experimental fibrosis in miR-155^−/−^ mice upon lung injury. A novel miR-155–regulated pathway identified in this context was the liver X receptor alpha (LXR)α, which is an oxysterol-activated transcription factor (NR1H3).^13^

## Methods

Bleomycin-induced lung fibrosis was induced in miR-155^−/−^ and control mice as described.^14^, ^15^ Mouse lung fibroblasts and macrophages were derived from wild-type (WT) and miR-155^−/−^ mice by lung digestion followed by fluorescence-activated cell sorting. Primary lung fibroblasts from patients with IPF (n = 7) and normal controls (n = 8) were obtained and cultured as described.^16^ Experimental interventions included transfecting cells with miR-155 mimic or LXRα siRNA, or incubating with LXR agonist/antagonist or various alarmins. Comprehensive details are provided in this article's Methods section in the Online Repository at www.jacionline.org.

## Results

### Experimental pulmonary fibrosis is exacerbated by miR-155 deficiency

**Fig 1:**
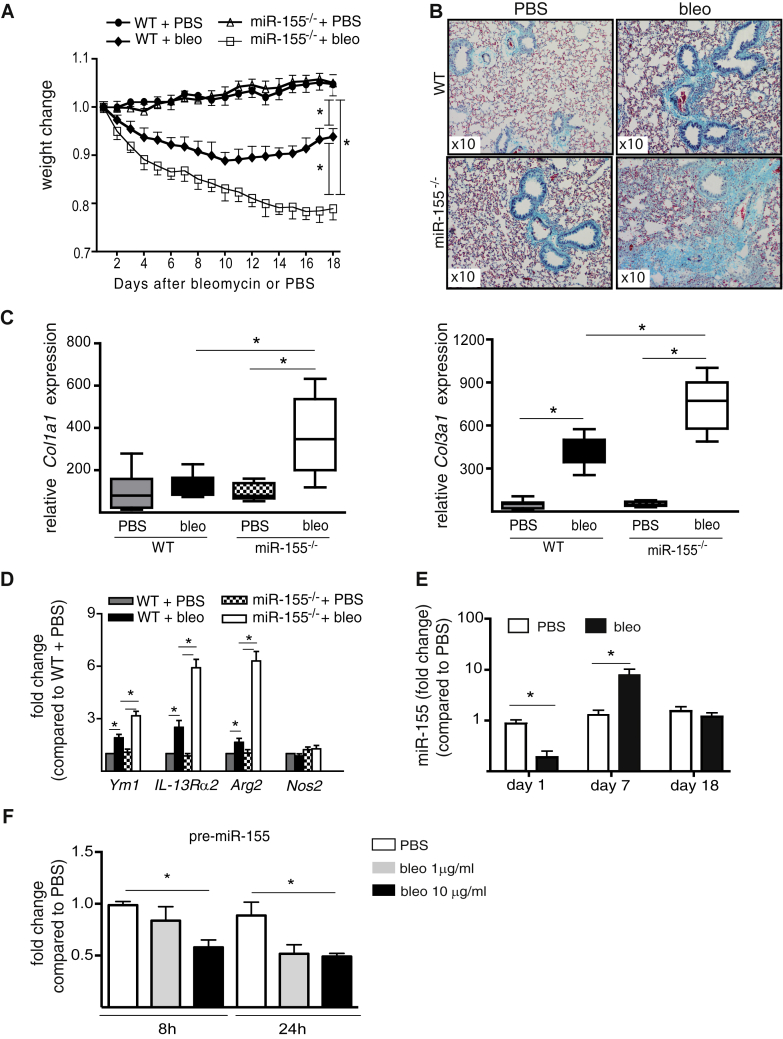
Deficiency of miR-155 exacerbates experimental model of pulmonary fibrosis. **A,** Bleomycin (1 dose intranasal) exacerbated a decrease in body weight. **B,** Lung collagen deposition (turquoise staining) in miR-155^−/−^ mice (n = 8). **C,** miR-155^−/−^ bleo mice show an increase in lung *Col1a1* and *Col3a1* mRNA. **D,** M2 macrophage polarization. **E,** miR-155 is dynamically regulated by bleomycin in WT mice. **F,** Precursor (pre-)miR-155 is decreased in lung fibroblasts of WT mice (pooled n = 5) cultured with bleomycin. *bleo*, Bleomycin. Data shown as mean ± SEM or median and interquartile range. *P < .05.

To evaluate miR-155 epigenetic control of lung fibrosis, we used the murine model of bleomycin-induced inflammation and pulmonary fibrosis.^17^ Bleomycin or control PBS was given to miR-155 gene-deleted (miR-155^−/−^) mice and WT controls. Bleomycin-induced weight loss (Fig 1, *A*), lung collagen deposition (Fig 1, *B*), and biomarkers of inflammation (see Table E1 in this article's Online Repository at www.jacionline.org) were exacerbated in miR-155^−/−^ mice compared with WT mice on day 18. This was accompanied by increased lung tissue expression of mRNA for collagen (*Col*)*1a* (mainly *Col1a1* isoform) and *Col3a1* (Fig 1, *C*; see Fig E1 in this article's Online Repository at www.jacionline.org), TGF-β (*Tgf-β*) expression, and lung collagen protein (Table E1). The increased bronchoalveolar lavage cell counts in bleomycin-treated miR-155^−/−^ mice (Table E1) were predominantly macrophages with the repair-associated, alternatively activated (M2) phenotype (Fig 1, *D*) confirmed by increased arginase 2 (*Arg2*), chitinase, and IL-13 receptor α2 expression, whereas the expression of the classically activated macrophage (M1) phenotype marker, inducible nitric oxide synthase (*Nos2*), remained unchanged. Together, these data demonstrate that miR-155 deficiency exacerbated the pulmonary fibrotic response to bleomycin.

We next investigated the kinetics of lung tissue miR-155 expression in WT mice given bleomycin (Fig 1, *E*). Expression of miR-155 in mice given PBS remained constant, whereas in response to bleomycin, miR-155 expression decreased at day 1, increased at day 7, and normalized by day 18. To investigate the factors that might regulate these changes, we established that bleomycin incubated *in vitro* with WT murine primary lung fibroblasts was sufficient to dose dependently downregulate the expression of precursor miR-155 at 8 hours (Fig 1, *F*) and mature miR-155 at 24 hours (see Fig E2, *A*, in this article's Online Repository at www.jacionline.org). To mimic the effect of exposure to cytokines generated in the damaged lung,^17^, ^18^ miR-155 expression was measured in WT murine primary lung fibroblasts incubated with exogenous alarmins IL-33,^17^ IL-25,^19^ IL-1α,^18^ or High mobility group box 1 (HMGB-1)^20^ released in response to injury. There was no change in response to IL-33, IL-25, or HMGB-1 (Fig E2, *B*), but IL-1α increased miR-155 expression (Fig E2, *C*). Thus, the dynamic expression of miR-155 *in vivo* may reflect a homeostatic role in inflammation and repair in response to tissue injury.

### Prediction analysis identified LXRα as an miR-155 target in the lung

Identifying mRNA targets under the epigenetic control of miR-155 was our strategy to identify cryptic pathways involved in lung fibrosis. We performed *in silico* analysis of predicted and validated conserved mouse and human miR-155 targets (TargetScan and miRTarBase) along with targets expressed in lungs or described in respiratory or fibrotic diseases (Ingenuity Pathway Analysis database). This integrated approach identified target mRNAs (Table E2), including hypoxia and TGF-β pathways,^2^, ^21^ among which we validated increased expression of *Hif1a*, *Tgfβr2*, and *Smad1* mRNA in lung tissue of miR-155^−/−^ mice given bleomycin (see Fig E3 in this article's Online Repository at www.jacionline.org). In addition to these recognized profibrotic pathways, we identified LXRα, which has not hitherto been described in lung fibrosis. LXRα has a conserved 3′UTR seed-region sequence (GCAUUAA) complementary to miR-155; therefore, we highlighted this as a potential novel pathway to pathogenic fibrosis and this provides the basis of our study.

### Endogenous miR-155 targets human LXRα

**Fig 2:**
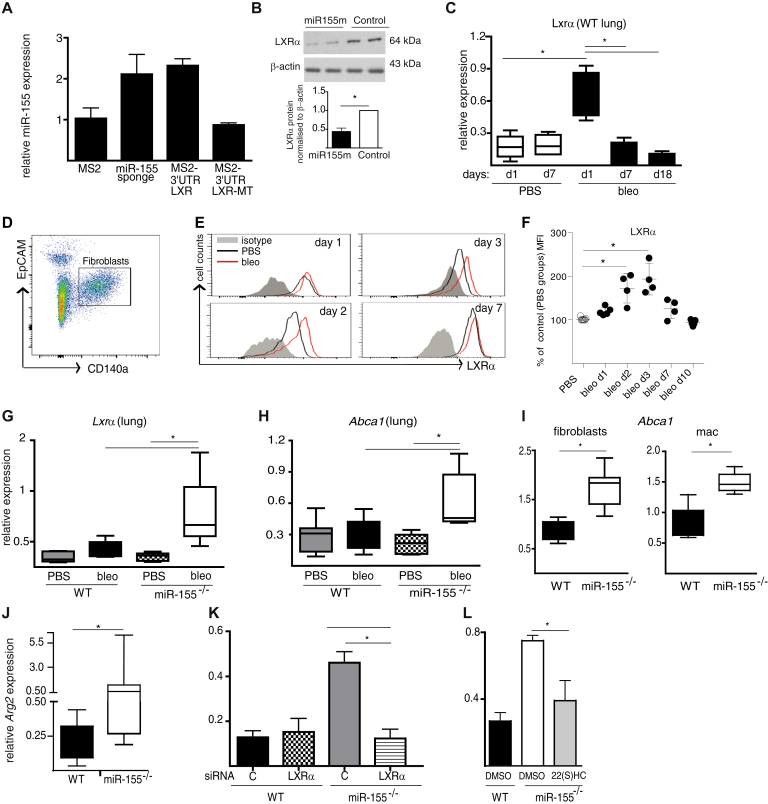
LXRα is regulated by miR-155. **A,** miR-155 binds to human LXRα. HEK293 were transfected with either empty vector (pmiRGLO-MS2BD) or miR-155 sponge (pmiRGLO-MS2BD-miR155Sp) or 3′UTR-LXRα (pmiRGLO-MS2BD-LXRα WT), or MS2 mutated in MRE 3′UTR-LXRα (pmiRGLO-MS2BD-Lxrα-MT), and miR-155 captured in the immunoprecipitate quantified by quantitative PCR. Data presented as mean ± SEM of 2 technical replicates; representative of 3 experiments. **B,** miR-155^−/−^ fibroblasts show downregulation of LXRα protein after transfection with miR-155 mimic. **C,** Time course of *Lxrα* mRNA expression in lungs of WT mice after bleomycin (n = 4-7 per group). **D,** Lung fibroblast gating strategy. Representative histograms **(E)** and quantitative evaluation **(F)** of an increase in LXRα expression in lung fibroblasts during fibrosis. Expression of *Lxrα***(G)** and *Abca1***(H)** in lungs of WT and miR-155^−/−^ mice on day 18. **I,** Constitutive expression of *Abca1* in lung fibroblasts (n = 4) and in alveolar macrophages (n = 4). Constitutive expression of *Arg2* in alveolar macrophages (n = 5) **(J)** and after transfection with Lxrα siRNA **(K)** or treatment with 22(S)HC (30 μM) **(L)**. Data presented as mean ± SEM or median and interquartile range. *bleo*, Bleomycin; *MRE*, microRNA recognition element; *DMSO*, dimethyl sulfoxide; *MS2BD*, MS2-binding domain. *P < .05.

We recently demonstrated using a reporter assay that synthetic miR-155 could bind mouse *Lxrα* mRNA.^22^ To confirm that endogenous miR-155 targets human *LXRα* mRNA, we used an MS2-TRAP RNA affinity purification assay.^23^ Expression constructs encoding luciferase genes tagged with the MS2-binding domain motif with either intact *LXRα*, or *LXRα* mutated in the 3′UTR microRNA recognition element as a negative control, were transfected into HEK293 cells together with the MS2GFP-expressing plasmid. The empty vector and a construct containing a tandem of 9 miR-155 binding sites (ie, an miR-155 “sponge”) were used as negative and positive controls, respectively. MS2-binding domain–containing transcripts were isolated from transfected cells by immunoprecipitation of green fluorescent protein and the enrichment of miR-155 in precipitates was measured by quantitative PCR (Fig 2, *A*). Transcripts containing WT *LXRα* 3′UTRs showed significant enrichment in miR-155 compared with the mutated sequence, which showed miR-155 levels similar to the empty vector control; thus, endogenous miR-155 could bind to human *LXRα* mRNA. To confirm and extend this observation, we reintroduced miR-155 into miR-155^*−/−*^ murine lung fibroblasts by transfection with a synthetic miR-155 mimic. After 24-hour culture, cytosolic LXRα protein concentrations (Fig 2, *B*) were reduced 60% by miR-155. Together, these findings support a functional interaction between miR-155 and *LXRa* mRNA.

### LXRα expression and activity are increased in miR-155^−/−^ mice with lung fibrosis

Compared with WT mice given PBS, the expression of *Lxrα* mRNA in lung tissue of WT mice given bleomycin was upregulated on day 1 and had normalized by day 7 (Fig 2, *C*). This increase was confirmed at the protein level in lung fibroblasts of WT mice given bleomycin, peaking at days 2 and 3 and normalizing to control PBS levels at day 7 (Fig 2, *D-F*; see Fig E4 in this article's Online Repository at www.jacionline.org). This *in vivo* expression pattern of *Lxrα* was reciprocal to that of miR-155 (Fig 1, *E*) in WT mice. Consistent with the homeostatic molecular interaction between miR-155 and *Lxrα* mRNA, miR-155^−/−^ mice given bleomycin maintained higher levels of lung *Lxrα* expression compared with WT mice (Fig 2, *G*). This increased expression was associated with an increase in *Lxrα* activity as measured by the expression of its specific functional reporter *Abca1* in lung tissue mRNA (Fig 2, *H*). Together, these data demonstrate that the lack of epigenetic homeostatic regulation in miR-155^−/−^ mice was associated with a sustained increase in *Lxrα* expression and activity in response to bleomycin.

### Serum concentrations of LXR oxysterol ligands are unchanged in experimental fibrosis

Oxidized derivatives of cholesterol, oxysterols, for example, 24(S) hydroxycholesterol and 27-hydroxycholesterol, are natural ligands that stimulate the expression and activation of LXRα.^24^ We showed previously that miR-155^−/−^ mice have higher serum cholesterol concentrations while on a high fat diet^22^; therefore, to test whether different oxysterol concentrations in miR-155^−/−^ mice treated with bleomycin were responsible for the *Lxra* activation and exacerbated lung fibrosis, we profiled serum oxysterols using mass spectrometry (Table E3). We found no differences between any of the known LXRα ligands,^25^, ^26^ suggesting that the increased activation of the *Lxrα* pathway in miR-155^−/−^ mice was due to normal activation of more available LXRα.

### miR-155^−/−^ lung fibroblasts and macrophages have an LXR-dependent profibrotic phenotype

We next investigated the role of LXR pathway activation in primary lung fibroblasts and alveolar macrophages. Compared with WT cells, miR-155^−/−^ fibroblasts and macrophages had greater and constitutive expression of the LXR*a* reporter gene, *Abca1* (Fig 2, *I*), suggesting that the LXRα pathway itself was constitutively activated. In miR-155^−/−^ macrophages, this was associated with an increased profibrotic (M2) phenotype characterized by increased expression of *Arg2*, a key enzyme controlling the bioavailability of hydroxyproline for collagen synthesis^27^ (Fig 2, *J*). We demonstrated that this increased *Arg2* expression in miR-155^−/−^ macrophages was restored to normal by *Lxrα-*siRNA (Fig 2, *K*; see Fig E5, *A*, in this article's Online Repository at www.jacionline.org) and by LXR antagonist 22(S)-hydroxycholesterol (22(S)HC)^28^ (Fig 2, *L*). To extend this to human cells, we investigated the regulatory interrelationship between *LXRα* and miR-155 in the expression of *ARG2* in human macrophages. Healthy human monocyte–derived macrophages were transfected with control siRNA or *LXRα* siRNA, each with miR-155 inhibitor or control inhibitor (Fig E5, *C*). To induce *LXRα* and *ARG2* expression, the cells were cultured with LXR agonist GW3965 or control dimethyl sulfoxide. The LXR agonist–induced increase in *ARG2* expression was further increased by inhibition of miR-155, and this increase was restored to normal by *LXRα*-specific siRNA (see Fig E6 in this article's Online Repository at www.jacionline.org). Together, these data suggest that LXRα*-*dependent regulation of *ARG2* was governed by miR-155 in human and mouse macrophages.

**Fig 3:**
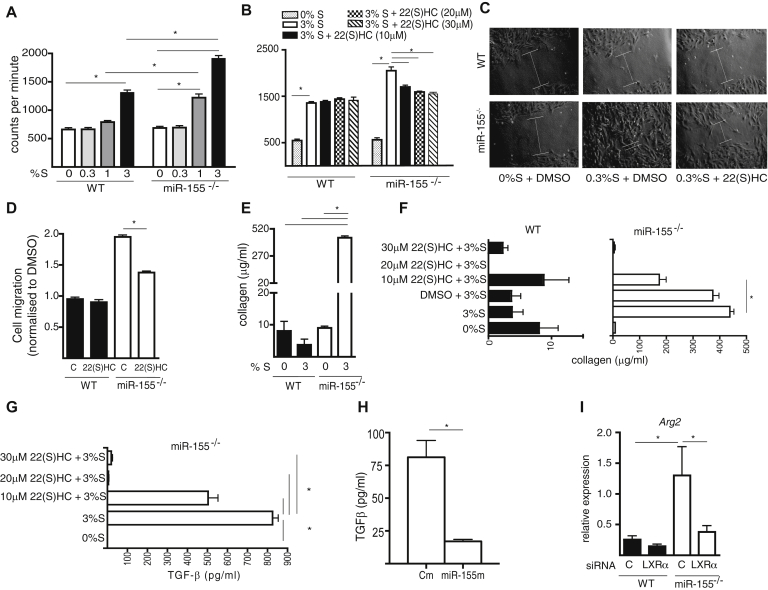
The phenotype of miR-155^−/−^ lung fibroblasts is driven by LXR. **A,** miR-155^−/−^ fibroblasts showed higher proliferation in response to FCS (%S) than did WT fibroblasts (pooled lungs of 4 mice). **B,** LXR antagonist 22(S)HC reduced the proliferation of miR-155^−/−^. **C** and **D,** miR-155^−/−^ fibroblasts had greater migration capacity; partially inhibited by 22(S)HC, and **(E)** produced more collagen than did WT fibroblasts. Collagen **(F)** and TGF-β production **(G** and **H)** by miR-155^−/−^ fibroblasts was inhibited by 22(S)HC (Fig 3, *G*) and by miR-155 mimic (Fig 3, *H*). **I,***Arg2* was higher in miR-155^−/−^ than in WT fibroblasts and this was reduced by LXRα siRNA. Data are presented as mean ± SEM of 4 biological replicates. *DMSO*, Dimethyl sulfoxide. *P < .05.

We next explored whether miR-155 influenced the profibrotic function of fibroblasts in an LXRα-dependent manner. *In vitro* proliferation, migration, and collagen production were compared in primary lines derived from mouse lung tissue. miR-155^−/−^ fibroblasts displayed greater proliferation to serum supplementation than did WT fibroblasts (Fig 3, *A*), which was restored to the normal proliferation observed in WT cells by the LXR antagonist 22(S)HC in a dose-dependent manner (Fig 3, *B*). miR-155^−/−^ fibroblasts also displayed increased migration compared with WT fibroblasts into the scratch space of an *in vitro* wound-healing assay, which was normalized by 22(S)HC (Fig 3, *C* and *D*). The increased fibroblast infiltration was not due to proliferation because the culture medium was supplemented with 0.3% FCS, a concentration that did not support fibroblast proliferation (Fig 3, *A*). miR-155^−/−^ fibroblasts produced approximately 40-fold increased concentration of soluble collagen in culture than did WT fibroblasts in response to 3% FSC (Fig 3, *E*), which was normalized in a dose-dependent manner by 22(S)HC to concentrations produced by WT fibroblasts (Fig 3, *F*).

TGF-β is the principal cytokine driving collagen gene expression, and oxysterol agonists of LXR can induce TGF-β production.^29^, ^30^ Therefore, to investigate the role of miR-155 in LXRα-dependent collagen production, we quantified TGF-β in WT and miR-155^−/−^ fibroblast supernatants cultured for 48 hours in 3% FCS, with/without 22(S)HC. miR-155^−/−^ fibroblasts produced higher concentrations of TGF-β than did WT fibroblasts and this increase was inhibited either by LXR antagonism (Fig 3, *G*) or by restoring miR-155 by transfection (Fig 3, *H*; see Fig E5, *B*). To investigate whether arginase was involved in this process, we measured the expression of *Arg2* in fibroblasts that were transfected with *Lxrα* siRNA or control siRNA (Fig E5, *B*). miR-155^−/−^ fibroblasts had higher expression levels of *Arg2* than did WT fibroblasts (Fig 3, *I*), and specific inhibition of *Lxrα* by siRNA restored *Arg2* expression in miR-155^−/−^ fibroblasts to the normal levels of WT fibroblasts. These observations indicate that excessive production of soluble collagen by miR-155^−/−^ fibroblasts may be due to an LXR-dependent increase in TGF-β and increased arginase-driven production of hydroxyproline.

### The exacerbated bleomycin-induced lung fibrosis in miR-155^−/−^ mice is LXR-dependent

**Fig 4:**
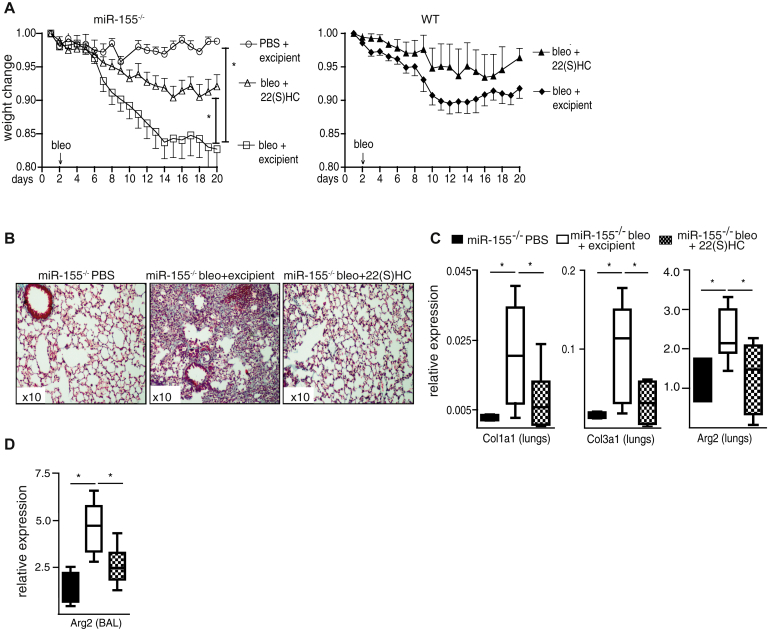
Inhibition of LXR ameliorates lung fibrosis in miR-155^−/−^ mice. From 2 days before the administration of bleomycin (n = 10), mice were treated with daily injections of 22(S)HC. Weight loss **(A)** and collagen deposition **(B)** (turquoise) in miR-155^−/−^ mice was mitigated by 22(S)HC. The expression of *Col1a1*, *Col3a1*, and *Arg2* in lung tissues **(C)** and *Arg2***(D)** in BAL cells in miR-155^−/−^ mice was reduced by 22(S)HC. Data presented as mean ± SEM or median (interquartile range). *BAL*, Bronchoalveolar lavage. *P < .05.

To test the involvement of LXR in experimental lung fibrosis, miR-155^−/−^ and WT mice were given bleomycin or control PBS, and treated with the LXR antagonist 22(S)HC or control cyclodextrin excipient. The subsequent loss of body weights for miR-155^−/−^ and WT mice is shown on different panels for clarity in Fig 4, *A*. The exacerbated bleomycin-induced weight loss in miR-155^−/−^ mice was mitigated by treatment with 22(S)HC to the weight loss seen in WT mice given bleomycin, as was the exacerbated lung tissue collagen deposition (Fig 4, *B*), and the inflammatory bronchoalveolar lavage cytology (see Fig E7 in this article's Online Repository at www.jacionline.org). The miR-155^−/−^–associated increased lung tissue *Col1a1*, *Col3a1*, and *Arg2*, and the bronchoalveolar lavage cell *Arg2* mRNA expression were also attenuated by 22(S)HC (Fig 4, *C* and *D*). 22(S)HC had no significant effect on weight loss in bleomycin-treated WT mice (Fig 4, *B*). These data demonstrate that the exacerbated inflammatory and fibrotic response to bleomycin in miR-155^−/−^ mice is at least partly dependent on LXRα and tractable *in vivo* by LXR antagonism.

### The exacerbated profibrotic behavior of IPF fibroblasts is normalized by neutralization of the LXR pathway

**Fig 5:**
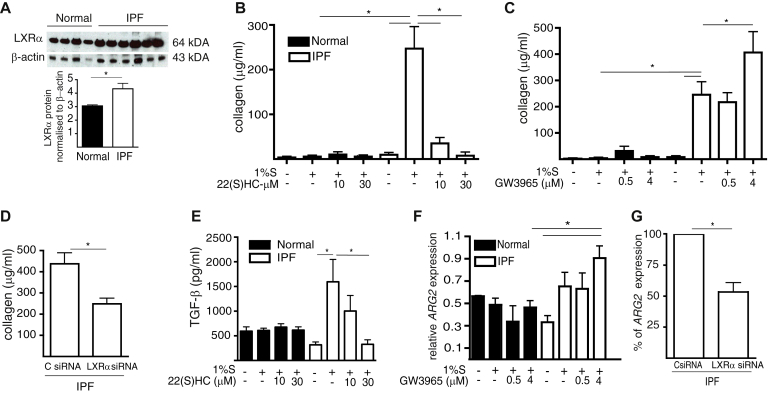
The profibrotic phenotype of IPF fibroblasts can be normalized by neutralization of LXR. Synchronized normal (n = 4) and IPF (n = 6) primary lung fibroblasts were cultured with 1% FCS (%S). **A,** IPF fibroblasts contained higher concentrations of LXRα protein. **B,** Collagen production by IPF fibroblasts was (Fig 5, *B*) reduced by 22(S)HC or potentiated by GW3965 **(C)**. **D,***LXRα* siRNA reduced collagen production by IPF fibroblasts. **E,** 22(S)HC inhibited TGF-β production. **F,** GW3965 potentiated serum-induced *ARG2* expression by IPF fibroblasts. **G,***Lxrα* siRNA reduced *ARG2* expression by IPF fibroblasts. Data are presented as mean ± SEM of 4 biological replicates. *P < .05.

To investigate the contribution of LXR pathway activation to the exacerbated lung tissue–remodeling characteristic of IPF, we obtained primary lung fibroblast lines from patients with IPF and control subjects (details in Tables E4 and E5). The constitutive cytosolic LXR*α* protein concentration was greater in IPF than in normal lung fibroblasts (Fig 5, *A*). IPF lung fibroblasts showed increased collagen synthesis *in vitro* compared with control lung fibroblasts, which could be either reduced in a dose-dependent manner by LXR antagonist (Fig 5, *B*) or further increased by the LXR agonist GW3965 (Fig 5, *C*). The contribution of LXR activation to the excess collagen production by IPF fibroblasts was further confirmed by transfecting IPF lung fibroblasts with *LXRα* siRNA (Fig E5, *D*), which attenuated the collagen production (Fig 5, *D*).

Control normal and IPF fibroblasts produced TGF-β in culture supernatants; however, only IPF fibroblasts increased TGF-β production in response to 1% FCS supplementation and this increased production was inhibited by LXR antagonist (Fig 5, *E*). Normal and IPF fibroblasts constitutively expressed similar levels of *ARG2* mRNA; however, only IPF fibroblasts showed higher expression of *ARG2* after stimulation with LXR agonist GW3965 (Fig 5, *F*) and this increased *ARG2* expression was attenuated by transfection with *LXRα* siRNA (Fig 5, *G*). These data indicate that TGF-β and ARG2 are regulated in an LXRα-dependent manner in IPF fibroblasts. In addition, compared with control lung fibroblasts, IPF lung fibroblasts showed greater *in vitro* proliferation in response to 1% FCS supplementation, which was reduced by LXR antagonist (see Fig E8, *A*, in this article's Online Repository at www.jacionline.org). IPF lung fibroblasts had increased migratory capacity into the scratch space of an *in vitro* wound-healing assay, compared with normal lung fibroblasts, and this increased migration was reduced to levels of normal fibroblasts by LXR antagonism (Fig E8, *B* and *C*). Thus, activation of the LXR pathway may drive the excessive profibrotic phenotypic characteristics of IPF fibroblasts.

### LXRα is deregulated from miR-155 in IPF lung fibroblasts

To test whether the LXRα-dependent collagen production by IPF fibroblasts was regulated by miR-155, control and IPF fibroblasts were transfected with miR-155 and stimulated by synthetic LXR agonist GW3965 *in vitro*. The increased collagen production by IPF fibroblasts was decreased (see Fig E9, *A*, in this article's Online Repository at www.jacionline.org), suggesting that collagen synthesis, as the prime exemplar of the LXRα-dependent profibrotic function of IPF fibroblasts, can be regulated by miR-155.

Because constitutively increased LXRα expression (Fig 5, *A*) and activity contributed to IPF fibroblast phenotype, we investigated whether this was caused by altered serum concentrations of LXRα oxysterol ligands in patients with IPF or by altered miR-155 expression. Comparing serum oxysterol concentrations in IPF and control subjects showed no differences in any of the LXRα ligands tested (Table E6). The constitutive miR-155 expression in IPF fibroblasts was similar to that of control lung fibroblasts (Fig E9, *B*); therefore, we investigated whether the increased LXRα expression and activation in IPF fibroblasts was due to a deregulated interaction between miR-155 and LXRα. Because the consequence of LXR*a* deregulation resulting in exacerbated lung fibrosis became apparent in miR-155^−/−^ mice only when stressed with bleomycin, we compared the dynamic interaction between miR-155 and LXRα in control and IPF fibroblasts cultured under the hypoxic stress (1% O_2_) that mimics the lung environment in IPF.^31^ Compared with normoxia, miR-155 expression was increased by hypoxia in both healthy and IPF fibroblasts (Fig E9, *C*); however, *LXRα* and *ABCA1* expression was increased by hypoxia only in IPF fibroblasts (Fig E9, *D*), suggesting selective deregulation of LXRα function. To explore the dynamics of the interaction between miR-155 and LXRα, we correlated the ratio of their relative expressions in normal and IPF lung fibroblasts. The relative expression levels of LXRα and miR-155 in normal and IPF lung fibroblasts cultured under normoxic conditions showed no significant correlation (Spearman *ρ* and 95% CI): normal fibroblasts *r* = 0.263 (−0.310 to 0.69) and IPF fibroblasts *r* = 0.439 (−0.072 to 0.767). However, under hypoxic conditions, there was a negative correlation in normal fibroblasts *r* = −0.655 (−0.868 to −0.236) that was not apparent in IPF fibroblasts *r* = −0.152 (−0.602 to 0.375) (Fig E9, *E*). This suggested that there was tight posttranscriptional control of LXRα expression by homeostatic miR-155 in response to a stressor such as hypoxia in normal fibroblasts that was lost in IPF fibroblasts, potentially contributing to the deregulated LXRα activity.

The mechanism of this deregulation may be due to increased competitive miR-155 binding by other mRNA targets that contain multiple miR-155 seed-region binding sites.^7^ To test this hypothesis, we evaluated the expression of a validated miR-155 target ZNF652^32^ that contains 7 miR-155 binding sites (HumanTargetScan v7.0) in normal and IPF fibroblasts cultured in normoxia and hypoxia. *ZNF652* was upregulated by hypoxia in IPF but not in normal lung fibroblasts (Fig E9, *F*) and in contrast to *LXRα*, the expression of *ZNF652* correlated negatively with miR-155 expression (Fig E9, *G*), suggesting that under hypoxic stress, miR-155 may be preferentially bound by the increased *ZNF652* leading to derepression of LXRα in IPF fibroblasts.

## Discussion

**Fig 6:**
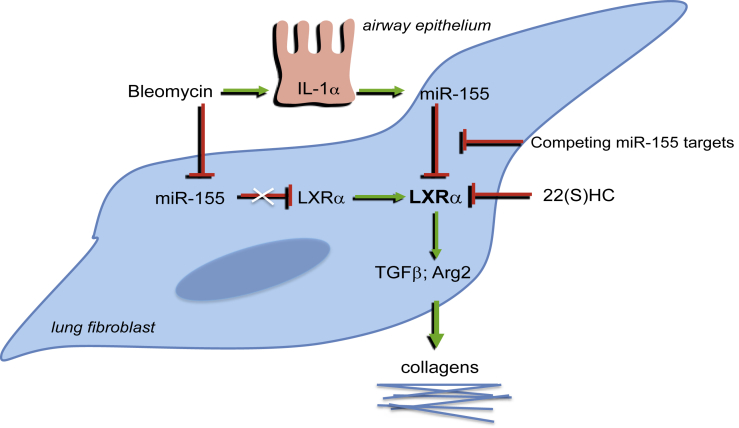
Deregulation of the miR-155/LXRα axis contributes to exacerbated pulmonary fibrosis. *TGF-β*, Transforming growth factor β.

Characteristic IPF fibrosis is refractory to anti-inflammatory therapy^4^ and antifibrotic drugs underline the primacy of aberrant wound healing to pathogenesis.^3^ We provide new understanding of this process. Mouse models and IPF lung fibroblasts had constitutively increased *LXRα* transcription when deregulated from homeostatic miR-155, associated with LXR-dependent excessive fibrotic phenotype mediated by increased TGF-β, arginase, and collagen production that could be mitigated by LXR antagonist (Fig 6).

Expression of miR-155 is rapidly and transiently reduced in WT mice after bleomycin, associated with transient reciprocally increased *Lxrα* expression and protein, and the remodeling is self-limiting.^15^ In contrast, miR-155^−/−^ mice have constitutively increased *Lxrα* and an exacerbated lung fibrosis, and this difference may provide novel insight into mechanisms of relentless lung remodeling. IPF lung fibroblasts also have constitutively more LXRα protein (and upregulated *LXRα* and *ABCA1*: IPF data repositories GSE2052^33^ and GEOD-24206^34^), and greater LXR-dependent profibrotic activation that was normalized by miR-155 overexpression, *LXRa* gene silencing, or metabolic antagonism of LXRα activity using 22(S)HC.

LXR may exert profibrotic effects by inducing *Arg2* and *Tgfβ* expression. The *Arg2* promoter contains an LXR response element and is activated by LXR agonism in macrophages,^35^ and we extend this finding to mouse and human fibroblasts. *Arg2* is the mitochondrial form involved in hydroxyproline production and is essential for collagen biosynthesis. Upregulated *Arg2* in miR-155^−/−^ macrophages and fibroblasts is normalized by inhibition of *Lxrα* by siRNA, or its activity by metabolic antagonism. LXR may also exert profibrotic effects by similarly regulating TGF-β expression, and the excessively high concentrations of TGF-β produced *in vitro* by miR-155^−/−^ and IPF fibroblasts were normalized by LXR antagonism.

Our profibrotic LXR function in lung conflicts with the antifibrotic function of T0901317-LXR activation in skin during experimental systemic sclerosis model.^36^ This can be reconciled; synthetic ligand T0901317 locks LXR into the conformation that recruits coactivators, whereas natural oxysterol ligands and GW3965 induce the flexible conformation that binds coactivators and corepressors,^37^ and there are tissue-specific epigenetic changes in chromatin that determine LXR-driven gene expression.^38^ Furthermore, the multiple-dose bleomycin-induced skin fibrosis is driven by IL-6 from inflammatory macrophages inhibited by LXR activation,^36^ whereas, in contrast, our single-bolus bleomycin-induced lung fibrosis is associated with repair M2 macrophage activation (Fig 1, *D*^17^), which is enhanced by LXR activation.^35^, ^39^ Alveolar macrophages are uniquely enriched in genes of lipid metabolism that are cross-regulated by LXR, supporting their role in lung homeostasis.^40^

The cryptic involvement of LXRα in fibrosis became apparent when deregulated in miR-155^−/−^ mice plus the stressor of bleomycin. The mechanism of *LXRα* deregulation in IPF fibroblasts may be due to ineffective regulation by miR-155, which becomes apparent under hypoxic stress equivalent to the IPF lung environment.^31^ IPF and control lung fibroblasts had similar miR-155 expression when cultured under normal oxygen tensions. Under hypoxic conditions, the expression levels of miR-155 correlated negatively with *LXRα* in control lung fibroblasts, implying tight epigenetic control, whereas there was no equivalent engagement between miR-155 and *LXRα* in IPF fibroblasts, thus enabling continued *LXRα* autoactivation^41^ and profibrotic behavior. This deregulation might be mediated by several mechanisms,^38^ including competition for available miR-155 by other targets with the AGCAUUAA seed-region^7^ as validated in cancer cells.^42^ One strong miR-155 candidate target mRNA is *ZNF652*, which has 7 seed-region binding sites. *ZNF652* is induced by hypoxia in IPF but not normal fibroblasts. We identified that in contrast to *LXRα*, its increased expression negatively correlated with miR-155 in IPF fibroblasts, suggesting that ZNF652 mRNA competitively bound miR-155 leading to derepression of *LXRa*.

Expression of miR-155 has been identified as increased^43^ or reduced,^44^ and serum miR-155 levels were normal^45^ in IPF. This may reflect the dynamism of miR-155 expression in experimental IPF. In lung tissue, it is transiently downregulated by bleomycin (Fig 1, *F*) and TGF-β,^46^ and induced by inflammatory mediators, for example, IL-1α (Fig E2, *C*) or hypoxia,^47^ as a counterbalance mechanism regulating homeostatic lung tissue repair.

Fibrosis of the lung is a common comorbidity of systemic sclerosis. The pathogenesis and clinical features of the autoimmune and inflammation-driven lung pathology of systemic sclerosis differs from IPF^48^ and 2 recent studies describe a pathogenic role for miR-155 in the experimental skin and lung fibrosis associated with systemic sclerosis.^49^, ^50^ This reflects the dual role of miR-155 driving chronic inflammation–associated pathologies and resolving fibrosis that we found aberrant in IPF.

**Key messages**

• Deficiency of miR-155 exacerbates bleomycin-induced experimental pulmonary fibrosis.
• In the absence of miR-155 epigenetic control, LXRα activity is deregulated in mouse primary lung fibroblasts facilitating increased collagen and TGF-β production, and in macrophages enhancing alternative activation, each inhibited by LXR antagonism, LXRα gene silencing, or exogenous miR-155 mimic.
• The exacerbated bleomycin-induced pulmonary fibrosis in miR-155^−/−^ mice was mitigated *in vivo* by LXR antagonism.
• Primary IPF lung fibroblasts had constitutively raised LXRα, deregulated from miR-155, and their profibrotic phenotype was inhibited by LXR antagonism, LXRα gene silencing, or exogenous miR-155 mimic.

## Supplementary data

Online Repository text

Legends for Figures E1-E9

Fig E1

Fig E2

Fig E3

Fig E4

Fig E5

Fig E6

Fig E7

Fig E8

Fig E9
