# Supplementary material for: The role of microRNA-155/liver X receptor pathway in experimental and idiopathic pulmonary fibrosis
Source: J Allergy Clin Immunol. 2017 Jun;139(6):1946–56. doi: 10.1016/j.jaci.2016.09.021 (PMC5457127; doi:10.1016/j.jaci.2016.09.021)
Supplement: Online Repository text [file mmc1.docx]

**Mariola Kurowska-Stolarska et al**

**The role of microRNA-155/LXR pathway in experimental and Idiopathic Pulmonary Fibrosis**

**SUPPLEMENTARY MATERIALS AND METHODS**

**Mice**

C57BL/6 microRNA-155-negative (miR155^−/−^) mice were obtained from Jackson Laboratories. Wild-type (WT) control littermates from miR155^−/−^ mice were backcrossed from C57BL/6 mice ^1^. Mice were maintained in a pathogen-free facility. Experiments were performed according to UK Home Office guidelines (Animals Scientific Procedures Act 1986).

**Reagents and experimental solutions**

The synthetic LXR agonist GW3965 was provided by Schering-Plough Corporation (UK) and dissolved in DMSO (Riedel-de Haen) for *in vitro* studies. The LXR antagonist 22(S)-hydroxycholesterol, 22(S)HC (Sigma) is an enantiomer of the natural LXR ligand 22(R)HC and acts as an antagonist of LXR pathway function ^2^. For *in vitro* use the antagonist was dissolved in DMSO, and for *in vivo* use, in a 40% solution of 2-hydroxypropyl-β-cyclodextrin in water; and the cyclodextrin was used as excipient control. Bleomycin sulphate, from *Streptomyces verticillus* (Sigma) was dissolved in PBS at a stock 1mg/ml concentration.

**Experimental pulmonary fibrosis**

Bleomycin administration generates a well-established murine model of pulmonary fibrosis ^3^ ^4^. One dose (0.06units/30μl/25g mouse) or control PBS was administered intranasally under light anesthesia ^5^. Daily control excipient 2-hydroxypropyl-β-cyclodextrin (40%) or therapeutic LXR antagonist 22(S)HC (*in vivo* t_1/2_ 8h ^2^) i.p. at 30 mg/kg were started two days prior. 7-10 mice per group were used per experiment.

**Endpoints used to assess experimental fibrosis**

The mice were monitored daily for wellbeing and changes in body weight. Serum, broncho-alveolar lavage (BAL) fluid, and lung tissues were harvested typically at day 18, and on other days as indicated, and analyzed as described previously ^6, 7^. Lung tissue was harvested and one lobe was collected into RNAlater, one lobe stored frozen for collagen content, and one lobe collected into 4% neutral buffered formalin and processed within 24h for histology. The extent of lung tissue inflammation and fibrosis was assessed by histological analysis of tissue sections stained with haematoxylin and eosin (H&E) and Masson’s Trichrome respectively. Two observers blinded to the nature of the samples scored the sections independently. Each scored the inflammation and fibrosis in 10 different microscope low-power fields per slide. The assessment of inflammation in each field was scored as follows: 1=histologically normal; 2=mild, 3=moderate and 4=severe inflammatory infiltrate; 5=complete loss of lung architecture. The inflammation score for each tissue section was the mean of the scores for each field. To evaluate the extent of the collagen deposition, a similar arbitrary score for fibrosis was as follows: 1=histologically normal; and 2=mild, 3=moderate and 4=severe collagen deposition across the parenchyma. The fibrosis score for each tissue section consisted of the mean of the scores for each field. Cytokine concentrations in serum and BAL fluid were measured using a 20-plex multi-cytokine assay (Life Technology) using a Luminex platform (Bio-Rad), and TGFβ1 was quantified using paired antibodies (R&D systems). Acid/pepsin soluble collagen, appropriate for lung soft-tissue or cell culture was measured by quantitative Sirius Red binding (Sircol assay, Biocolor).

**Human lung fibroblasts**

Primary fibroblast lines were cultured from explanted lung tissues from idiopathic pulmonary fibrosis (IPF) and normal subjects obtained with informed consent under a protocol approved by the University of Pittsburgh Institutional Review Board ^8^ (details on Tables E4 and E5).

**Fibroblast cell cultures**

Lung fibroblast lines (IPF n=7 and control normal n=8) were cultured as previously described ^9^. Fibroblasts from pooled lungs of WT (n=4-5) and miR-155^-/-^ (n=4-5) mice were isolated and cultured as described ^10^. For functional experiments, sub-confluent cells in culture were first synchronised in serum-free DMEM medium for 24h followed by incubation with medium containing different concentration of fetal calf serum (FCS; 0.3%-3%) for 48h. Different fibroblast functions were best demonstrated at different FCS concentrations; and pilot titration experiments showed that the optimum FCS (%) in WT and miR-155^-/-^ mouse lung fibroblasts for proliferation was (1 to 3%), for collagen production (3%) and for cell migration (0.3%). Similarly, for IPF and control lung fibroblast functions: for proliferation (3%), collagen production (3%) and migration (0.3%). Experiments were performed in 24 well plates, at a cell confluency of 70-80%. In some experiments LXR agonist GW3965 (0.5 μM, 4 μM) or LXR antagonist 22(S)HC (10, 20, 30 μM) or IL-33, IL-1α, IL-25, HMGB1 (10-100 ng/ml, all from Peprotech) or bleomycin (1, 10 μg/ml, Sigma) or bleomycin excipient (DMSO) were added. For proliferation assays, cells were pulsed with (^3^H) thymidine 4 hours before cell harvest. Control and IPF fibroblasts were tested typically in-group numbers of 2 each in several independent experiments. Experiments were performed in standard condition (normoxia, 21% oxygen) or in hypoxia (1% oxygen). Each variant of stimulation was typically done in 3 to 4 technical replicates.

**Evaluation of LXRα protein expression in lung cells by flow cytometry**

Wild type mice were treated with Bleomycin (n=24) with dose of 0.06 U per 25 g mouse in 30 μl pro-rata or PBS (n=7) by intranasal installation on day 0 and then lungs were isolated on days 1, 2, 3 7 and 10. Mouse lungs were digested in RPMI containing Dispase (3.2 mg/ml, Roche), Collagenase P (0.4 mg/ml, Roche) and DNAse I (0.2 mg/ml) in 37^0^C with rotation for 1h. Digests were filtered through 100 μm strainer and stained with Fixable Viability Dye eFluor® 780, 65086514, eBioscience) followed by the incubation with the antibodies against CD45 (109822), EpCAM (118220), CD141a (135908) and F4/80 (123110, all from BioLegend) ^11^. After 30 minutes, cells were washed and fixed/permeabilised with Transcription Factor Staining Buffer set (00552300, eBioscience) followed by incubation with anti-LXRα antibody (ab176323, Abcam) and Brilliant Violet 421™ Donkey anti-rabbit IgG Antibody (406410; BioLegend). LXRα protein expression was then evaluated in different lung cell types (fibroblasts, macrophages and epithelial cells). The gating strategy is shown on Fig, E1. Median fluorescence intensity (MFI) of isotype control was subtracted from MFI of LXRα and presented as a % of MFI of PBS group.

**Manipulation of expression of miR-155 and LXRα.**

To evaluate the contribution of LXRα to activation of fibroblasts, cells were transfected with LXRα-specific siRNA or All Stars Negative Control siRNA unlabeled or labeled with Alexa Fluor 488 (50 nM, both from Qiagen) using DharmaFECT 3 transfection reagent (Thermo Scientific). 16h later, cells were stimulated with medium containing 1% FSC or synthetic LXR agonist GW3965 for a further 24h. To investigate the effect of miR-155 on fibroblast function, cells were transfected with mouse or human miR-155 mimic or control nucleotide mimic (*C. elegans* miR-67) unlabeled or labeled with Dy-547 (40 nM, both from Dharmacon). 16h later, the cells were stimulated with medium containing 1% FCS or supplemented with LXR agonist as described above. In all experiments, the transfection efficiency of siRNA and microRNA mimic was checked by flow cytometry; and only when more than 60% of cells were positive were they used for further analysis. Cells were collected for RNA and the culture supernatants collected for assay of soluble collagen and TGFβ.

**Cell migration measured by *in vitro* wound-healing scratch assay**

To evaluate the cell migration consistent with the wound healing properties of fibroblasts, an *in vitro* scratch assay was performed as described previously ^12^. Briefly, cells were allowed to grow into a confluent monolayer and a single uniform scratch was applied to the cell layer in each well using a 100-μl pipette tip. The cells were allowed to settle for 3h and then treated with either: vehicle, 22(S)HC (30μM) in medium for the next 24h. The scratch was visualized using phase-contrast microscopy (×10 objective) and photographed, and the width of the wound was measured at 0 and 24h. A line was drawn along each side of the scratch to remove minor irregularities generated by the mechanical scratch from the pipette tip, and the distance between the lines measured at 4 separate points. This was quantified in arbitrary units describing the scratch closure; from 1 = completely open scratch to 2 = total closure. Each variant of stimulation was done in 3 to 4 technical replicates.

**Human monocyte-derived macrophages**

CD14 positive cells were isolated from buffy coats using microbeads (Militenyi Biotech). Cells were differentiated into macrophages by incubation with M-CSF (50ng/ml) in RPMI 1640/10%FCS. After 6 days, macrophages (0.25 x10^6^/well in 24 well plates) were transfected (DharmaFECT3) with human miR-155 inhibitor or control inhibitor (40 nM, Qiagen). 16h later the culture medium was changed and cells were transfected (DharmaFECT3) with LXRα siRNA or control siRNA (50nM, Qiagen). Cells were harvested 24h later and RNA was purified with miRNeasy kit (Qiagen). Each variant of stimulation was done in technical replicates (n=3-4).

**Mouse alveolar macrophage culture**

Cells obtained by bronchoalveolar lavage (BAL) from WT (n=11) and miR-155^-/-^ (n=11) mice were counted and seeded in 200 μl of RPMI1640/10%FSC (0.1 x10^6^/well in 96-well plates). After 24h incubation, non-adherent cells were washed away and adherent alveolar macrophages were harvested for RNA isolation (n=5) or transfected with LXRα siRNA or control siRNA, n=3 (50 nM, Qiagen). In some experiments, alveolar macrophages (n=3) were incubated with DMSO or 22SHC (30μM). Cells and supernatants were collected 24h later.

**QPCR**

Total RNA, including the small RNA fraction was isolated using the miRNeasy kit (Qiagen). cDNA was prepared using either the TaqMan microRNA Reverse Transcription (RT) kit or the high capacity cDNA RT kit (both from Life Technologies). TaqMan mRNA or microRNA assays (both from Life Technologies); or pairs of specific primers (Integrated DNA Technologies) in conjunction with SYBRgreen method (Life Technologies) were used for semi-quantitative determination of the expression of human and mouse microRNAs and mRNAs. Details of the sequence and name of the assays are provided in Table E7. Expression of RNU6 snRNA or 18S or β-actin or TBP (TATA binding protein) was used as endogenous control. Gene expression was analysed on an Applied Biosystems ABI7900HT machine with SDS 2.2 software. Data is presented as relative values (2^-ΔCT^) with the expression relative to endogenous control; RNU6 for miR-155; 18S or β-actin for human mRNAs and 18S or TBP for mouse mRNAs. In some cases data are presented as fold change compared to control sample (2^-ΔΔCT^).

**Western blot for LXRα**

Sub-confluent normal and IPF lung fibroblast primary cell lines in culture were synchronised for 24h in serum-free medium and then cultured for 24h in medium supplemented with 1% FCS. Similarly, miR-155^-/-^ murine lung fibroblasts were synchronised for 24h and then transfected with control (*C. elegans* miR-67) or mouse miR-155 mimic (40nM; both from Dharmacon) in medium containing 1% FCS. After 24 hours, whole cell lysates were prepared using M-PER (Thermo Scientific) lysis buffer. Total protein (10µg) was applied to and run on a 10% SDS Page gel followed by transfer to PVDF membrane, and incubation with rabbit anti-mouse/human LXRα (2µg/ml; Lifespan Biosciences) or anti-mouse/human β-actin (1µg/ml, Santa Cruz Biotechnology).

**MS2 pull-down assay**

MS2-TRAP RNA affinity purification was performed as described in Yoon et al ^13^. Briefly*,* pmiRGLO vector (Promega) was modified to incorporate an MS2 binding domain (MS2BD) to create pmiRGLO-MS2BD. The 3’UTR from the human *LXRα* gene containing the predicted miR-155 binding sites were cloned downstream of the luciferase open reading frame (ORF) and MS2BD, creating pmiRGLO-MS2BD-Lxrα (WT). pmiRGLO-MS2BD-Lxrα (MT) in which the seed-region of the predicted miR-155 MRE was mutated using Quick Change Lightning site-directed mutagenesis kit (Agilent) was used as a negative control. pmiRGLO-MS2BD-155 sponge (MS2BD-155s), containing 9 miR-155 binding sites, was synthesised to order from Integrated DNA Technology and cloned downstream of luciferase ORF in pGLO-MS2BD. This was used as a positive control. 1 million HEK293 cells were transfected with 1μg pMS2GFP plus 1μg of the relevant pmiRGLO-MS2BD vector using Lipofectamine 2000 (Invitrogen). Cells were grown for 48 hours. MS2GFP complexes were immuno-precipitated using anti-GFP IP kit (Miltenyi) supplemented with RNAase inhibitor (10U/ml) (Invitrogen). Precipitated RNAs were purified using miRNEASY kit (Qiagen). cDNA was synthesised using miRSCRIPT II kit (Qiagen) with HiFlex buffer. miR-155 was measured by qPCR using miR-155 Primer assay kit (Qiagen). miR-155 expression was normalised to luciferase transcript levels and expressed as relative expression to pmiRGLO-MS2BD empty vector.

**Oxysterol assay**

**The serum samples from IPF patients and healthy control subjects** was obtained and approved by the Lothian Research Ethics Committee. Oxysterols in these and mouse serum samples were analysed as described in Griffiths et al. ^14^. Briefly, sterols and oxysterols were extracted with ethanol and separated according to hydrophobicity on a reversed phase Oasis HLB column. The oxysterol fraction was split into two sub-fractions. Fraction A was treated with cholesterol oxidase followed by Girard P (GP) reagent, while fraction B was derivatised with GP reagent directly in the absence of enzyme. This allows the separate detection of oxysterols with and without an oxo group, respectively. The GP derivatised oxysterols were then separated from excess derivatisation reagent on a C_18_ column and analysed by liquid chromatography – mass spectrometry (LC-MS) with multistage fragmentation (MS^n^). Oxysterols were identified by retention time, exact mass (5 ppm) and MS^n^ by reference to authentic standards. Quantification was by isotope dilution mass spectrometry.

**Statistical analysis**

Data was analyzed by GraphPad prism or Minitab. Between-category differences in biomarker concentrations and gene expression levels were compared by Mann-Whitney *u*-test or one-way Anova with Tukey’s multiple comparison tests Results from related cell cultures were compared by paired-*t*-test. Data illustrated as mean ± SEM, or box-and-whisker and dot-plot plots with medians and means respectively. Mass-spectrometry data presented as mean±SD. *P*<0.05 was considered statistically significant.

**SUPPLEMENARY TABLES**

|  | **PBS** | | |  | **Bleomycin** | | |
| --- | --- | --- | --- | --- | --- | --- | --- |
|  | **WT** | **miR-155^-/-^** | **p** |  | **WT** | **miR-155^-/-^** | **p** |
| **Lung histology**  fibrosis score  inflammation score | 1.20 (1.10, 1.30)  1.22 (1.13, 1.35) | 1.20 (1.15, 1.30)  1.27 (1.21, 1.52) | 0.698  0.406 |  | 2.79 (2.27, 2.99)  2.44 (2.21, 2.91) | 3.10 (2.69, 3.59)  3.80 (3.26, 4.15) | 0.037  0.001 |
| **Lung lavage**  cell count x10^6^  macrophage x10^6^  lymphocytes x10^4^ | 0.10 (0.10, 0.20)  0.10 (0.09, 0.19)  0.07 (0.03, 0.10) | 0.20 (0.10, 0.40)  0.10 (0.09, 0.30)  0.16 (0.04, 0.55) | 0.178  0.443  0.096 |  | 1.33 (1.20, 1.44)  0.92 (0.90, 1.08)  2.6 (0.44, 4.4) | 7.55 (6.42, 8.15)  4.52 (3.09, 5.97)  23.3 (18.9, 31.1) | 0.001  0.001  0.001 |
| **Lung tissue**  TGFβ mRNA *(tgfb)*  collagen (μg/g) | 0.98 (0.93, 1.07)  2.00 (1.87, 2.23) | 0.98 (0.95, 1.06)  2.19 (1.93, 2.30) | 1.000  0.523 |  | 3.96 (2.94, 5.34)  2.60 (2.43, 2.91) | 6.25 (5.05, 7.99)  2.90 (2.73, 3.35) | 0.024  0.016 |

**Table E1. Comparison of biomarkers of lung fibrosis and inflammation between wild-type (WT) and miR-155^-/-^ mice given control PBS or bleomycin**. Lung tissue and lavage were harvested 18 days after PBS or bleomycin. Inflammation and fibrosis was scored histologically (Methods in this article online repository). Lavage cytology was determined by total and differential cell counting of H&E stained cyto-centrifuge slide preparations. Expression of TGFβ mRNA in lung tissue was determined by qPCR. Lung collagen was quantified by Sircol assay. Median (IQR), with Mann-Whitney p-values.

The following cytokines were detectable in BAL fluid (TNF-α, IL-1β, IL-4, IL-10, IL-12, CXCL10, CXCL1, CXCL9, CCL3, basic FGF, VEGF), and in serum (TNF-α, IL-1α, IL-1β, IL-4, IL-5, IL-10, IL-12, CXCL10, CXCL1, CXCL9, CCL3, basic FGF, VEGF, TGFβ, IFN-γ, CCL2). The concentrations of VEGF and CXCL1 in serum and bFGF in BAL were changed by bleomycin compared with PBS treatment, however none were changed further in miR-155^-/-^ mice compared with WT mice both given bleomycin. The following cytokines were undetectable in BAL fluid (GM-CSF, IFN-γ, IL-1α, IL-2, IL-5, IL-6, IL-13, IL-17, CCL-2), and in serum (GM-CSF, IL-2, IL-6, IL-13, IL-17).

| **Target** | **Name** | **Reference** |
| --- | --- | --- |
| AGTR1 | Angiotensin II receptor, type 1 | Martin MM ^15^ |
| SMAD1  SMAD5 | SMAD* family member 1  SMAD family member 5 | Rai D ^16^ |
| SMAD2 | SMAD family member 2 | Louafi F ^17^ |
| HIF-1α | Hypoxia-inducible factor 1-alpha | Bruning U ^18^ |

**Table E2. Conserved and validated miR-155 targets that are expressed in lungs.** The stepwise prediction-target identification strategy identified LXRα among experimentally validated miR-155 targets such as AGTR1, HIF-1α, BMP and TGFβ signalling molecules; SMAD2, SMAD1 and 5, as potentially targets involved in miR-155 fine-tuning of the remodeling process in the lung. Among them however only HIF-1α and SMAD5 are miR-155 conserved target in mouse and human. We confirmed that HIF-1α mRNA was a target by demonstrating that its expression was increased in lung tissue of miR-155^-/-^ mice given bleomycin (Fig. E3) compared to WT or PBS controls, as well as confirming the integrity of the miR-155 gene deleted mouse. *SMAD is an acronym for protein homologs of *Drosophila* protein (mothers against decapentaplegic; MAD) and *C. elegans* protein SMA (gene *sma*; small size).

|  | **Mean concentration ng/mL** | | | | | | | |
| --- | --- | --- | --- | --- | --- | --- | --- | --- |
|  | **PBS treatment** | | | | **Bleomycin treatment** | | | |
|  | **Wild type** | | **miR-155^-/-^** | | **Wild type** | | **miR-155^-/-^** | |
| **Sterol Systematic Name (Common name)** | **Mean** | **SD** | **Mean** | **SD** | **Mean** | **SD** | **Mean** | **SD** |
| 7α-Hydroxy-3-oxocholest-4-en-26-oic acid^6^ | 38.18 | 19.41 | 51.84 | 27.68 | 20.86 | 1.95 | 31.35 | 3.45 |
| 7α-Hydroxycholest-4-en-3-one | 14.93 | 6.18 | 16.87 | 12.73 | 12.46 | 3.11 | 12.36 | 3.28 |
| 3-Oxocholesta-4,6-dien-26-oic acid^6^ | 8.11 | 6.85 | 11.52 | 9.71 | 3.84 | 1.86 | 3.10 | 1.34 |
| Cholest-5-ene-3β,7α-diol (7α-Hydroxycholesterol)^5^ | 5.04 | 4.62 | 11.91 | 20.62 | 2.55 | 1.28 | 2.19 | 1.51 |
| 3β-Hydroxycholest-5-en-7-one (7-Oxocholesterol)^5^ | 4.09 | 3.83 | 1.40 | 1.74 | 3.52 | 1.70 | 2.91 | 1.61 |
| Cholest-5-ene-3β,7β-diol^4^ (7β-Hydroxycholesterol) | 3.81 | 3.35 | 2.90 | 0.80 | 2.66 | 0.50 | 1.44 | 0.66 |
| 3β-Hydroxycholest-5-en-26-oic acid^3^ | 2.16 | 0.35 | 1.93 | 0.24 | 3.12 | 0.49 | 3.02 | 0.42 |
| Cholest-5-ene-3β,24S-diol^3^ (24S-hydroxycholesterol) | 1.78 | 0.25 | 1.18 | 0.41 | 1.86 | 0.94 | 1.08 | 0.43 |
| 3β,7α-Dihydroxycholest-5-en-26-oic acid^7^ | 1.37 | 1.34 | 0.81 | 1.63 | 0.82 | 0.47 | 1.29 | 1.47 |
| 7α,26-Dihydroxycholest-4-en-3-one | 1.30 | 0.41 | 1.91 | 0.96 | 0.76 | 0.17 | 0.96 | 0.17 |
| 7α,25-Dihydroxycholest-4-en-3-one | 1.14 | 0.31 | 0.97 | 0.44 | 1.02 | 0.67 | 0.83 | 0.15 |
| 7α-Hydroxy-26-nor-cholest-4-ene-3,24-dione^1,2^ | 1.10 | 0.48 | 1.31 | 0.29 | 1.88 | 0.46 | 2.54 | 1.45 |
| 3β,7β-Dihydroxycholest-5-en-26-oic acid^3^ | 0.95 | 0.18 | 0.88 | 0.20 | 0.87 | 0.32 | 1.13 | 0.17 |
| Cholest-5-ene-3β,26-diol^3^ ((25R),26-Hydroxycholesterol) | 0.83 | 0.42 | 0.56 | 0.45 | 0.87 | 0.90 | 0.48 | 0.47 |
| Cholest-5-ene-3β,25-diol^3^ (25-hydroxycholesterol) | 0.56 | 0.58 | 0.18 | 0.15 | 0.30 | 0.39 | 0.17 | 0.12 |
| 3-Oxocholest-4-en-26-oic acid | 0.55 | 0.23 | 0.56 | 0.32 | 0.64 | 0.20 | 0.62 | 0.12 |
| 3β,22,25-Trihydroxycholest-5-en-24-one^1^ | 0.38 | 0.30 | 0.53 | 0.47 | 0.43 | 0.44 | 0.44 | 0.33 |
| Cholest-5-ene-3β,7α,25-triol (7α,25-Dihydroxycholesterol) | 0.04 | 0.04 | 0.15 | 0.12 | 0.07 | 0.08 | 0.02 | 0.04 |
| Cholest-5-ene-3β,7α,26-triol (7α,26-Dihydroxycholesterol) | 0.01 | 0.02 | 0.03 | 0.05 | 0.03 | 0.04 | 0.00 | 0.00 |
| 3β-Hydroxycholesta-5,7-dien-26-oic acid^7^ | 0.00 | 0.00 | 0.00 | 0.00 | 0.07 | 0.14 | 0.09 | 0.16 |

**Table E3. Oxysterols and cholestenoic acids in mouse serum after control PBS or bleomycin treatment.** Oxysterols and cholestenoic acids identified by LC-ESI-MS^n^ in serum following solid phase extraction and charge-tagging with GP-hydrazine. In the absence of authentic standards presumptive identifications based on exact mass, MS^n^ spectra and retention time are given. Quantitation was by stable isotope dilution. Samples from four PBS-treated wild type and miR-155^-/-^ mice and four bleomycin-treated wild-type and miR-155^-/-^ mice were analysed. We have adopted the systematic sterol nomenclature recommended by the Lipid Maps consortium http://www.lipidmaps.org/. In this nomenclature hydroxylation of the terminal carbon of the sterol side-chain introducing R stereochemistry at C-25 is defined as C-26 hydroxylation.  ^1^ Presumptive identification based on exact mass and MS^n^ spectra. ^2^ 26-Nor-sterol is a likely decomposition product of a 24-oxo-26 acid. ^3^ LXR ligand. ^4^ May be formed by autoxidation. ^5^ Can be formed enzymatically and by autoxidation. ^6^ 7α-Hydroxy-3-oxocholest-4-en-26-oic acid dehydrates to a minor degree to 3-oxocholesta-4,6-dien-26-oic acid. Thus, the total 7α-hydroxy-3-oxocholest-4-en-26-oic acid corresponds to the sum of the two acids. ^7^ 3β,7α-Dihydroxycholest-5-en-26-oic acid dehydrates to a minor degree to 3β-hydroxycholesta-5,7-dien-26-oic acid. Thus, the total 3β,7α-dihydroxycholest-5-en-26-oic acid corresponds to the sum of the two acids. To be noted 27-hydroxycholesterol is (25R)26-hydroxycholesterol according to IUPAC nomenclature.

| IPF-  Code | age | sex | race | smoke | onset | FVC | FEV1 | TLC | DLCO | EF | PA echo | PA cath | PA mean | PA  WP | 6-min  walk |
| --- | --- | --- | --- | --- | --- | --- | --- | --- | --- | --- | --- | --- | --- | --- | --- |
| 117 | 71 | f | h | 0 | n.a. | 51 | 54 | n.a. | 29 | 55 | 39 | 27/9 | 17 | 6 | 610 |
| 124 | 64 | m | c | 60 | n.a. | 67 | 80 | n.a. | 37 | 60 | 39 | 30/14 | 21 | 7 | 1430 |
| 131 | 63 | f | c | 0 | 5 | 57 | 66 | 60 | 15 | 55 | 71 | 70/21 | 40 | 10 | 470 |
| 133 | 61 | f | a | 0 | 10 | 20 | 23 | n.a. | n.a. | 55 | 20 | 45/8 | 30 | 10 | 550 |
| 135 | 67 | f | c | 30 | < 5 | 60 | 64 | 67 | 26 | 65 | 46 | 44/17 | 27 | 10 | 60 |
| 136 | 75 | f | c | 0 | 3 | 57 | 67 | 55 | 26 | 60 | 46 | 39/14 | 23 | 12 | 900 |
| 161 | 65 | f | c | 50 | n.a. | 48 | 64 | n.a. | n.a. | 55 | 46 | 48/22 | 31 | 20 | 280 |

**Table E4. Clinical and demographic details of IPF patients.** IPF code I.D. number, Age (years), Gender (f=female, m=male), race (h=Hispanic, c=Caucasian, a=Arabic/middle-eastern), smoking history (pack years), onset of symptoms (years), lung function; % of predicted normal: Forced Vital Capacity (FVC), Forced Expiratory Volume in one second (FEV1), Total Lung Capacity (TLC), Extinction Fraction (EF), Pulmonary Artery echo, pressures and WP, and 6-minute walk test (feet). No patient was taking therapy for pulmonary hypertension. All patients were taking immune-suppressive therapy (prednisolone plus mycophenolate mofetil).

| NL-  Code | age | sex | race | smoke | Cause of Death | Notes |
| --- | --- | --- | --- | --- | --- | --- |
| 51 | 76 | f | n.a. | 0 | CVA | mild pulmonary edema on CXR |
| 57 | 60 | f | n.a. | 0 | n.a. | On vent 1+ days, patchy infiltrates sparing RLL, minimal secretions at Bronch. |
| 59 | 50 | f | n.a. | 0 | CVA; donated after cardiac death | Other lung was used for a single transplant; less than 1 hr of warm ischemia. |
| 60 | 60* | n.a. | n.a. | 0 | CVA after neurosurgery | basilar atelectasis |
| 62 | 62 | f | n.a. | 10 | intracerebral bleed after initial CVA | CXR clear; 10 years of diabetes; past history of resected squamous cell of nose; PO2 78 on 60% FiO2, likely related to atelectasis or transient arrest earlier today. On antibiotics so covered for aspiration at time of CVA. |
| 67 | 25 | m | n.a. | n.a. | GSW to the head | Mild basilar density c/w atelectasis or infiltrate |
| 71 | 33 | f | b | n.a. | Unknown | Autopsy |
| 74 | 37 | m | c | n.a. | Lung resection; benign granuloma | Surgery; not a transplant donor |

**Table E5. Clinical and demographic details of post-mortem donors of control lung tissue.** Normal lung code I.D. number, Age (years, *60=early 60’s), Gender (f=female, m=male), race (c=Caucasian, b=Black), smoking history (pack years), details of cause of death and clinical notes. n.a. = data not available.

| **­** | **Serum concentration ng/mL** | | | |
| --- | --- | --- | --- | --- |
|  | **Control n=6** | | **IPF n=9** | |
| **Sterol Systematic Name (Common name)** | **Mean** | **SD** | **Mean** | **SD** |
| 3β-Hydroxycholest-5-en-26-oic acid^3^ | 90.68 | 31.98 | 79.11 | 26.58 |
| 7α-Hydroxy-3-oxocholest-4-en-26-oic acid^6^ | 76.03 | 28.31 | 90.89 | 26.76 |
| 3β,7α-Dihydroxycholest-5-en-26-oic acid^3,7^ | 29.51 | 17.03 | 45.29 | 19.04 |
| Cholest-5-ene-3β,26-diol^3^ ((25R),26-Hydroxycholesterol) | 24.36 | 3.85 | 21.88 | 11.55 |
| 3β-Hydroxycholest-5-en-7-one^5^ (7-Oxocholesterol) | 22.46 | 23.01 | 68.03 | 112.00 |
| 7α-Hydroxycholest-4-en-3-one | 18.55 | 16.48 | 15.52 | 11.52 |
| Cholest-5-ene-3β,7α-diol^5^ (7α-Hydroxycholesterol) | 17.31 | 9.19 | 22.14 | 27.06 |
| Cholest-5-ene-3β,7β-diol^4^ (7β-Hydroxycholesterol) | 15.91 | 18.03 | 19.21 | 29.42 |
| 3-Oxocholesta-4,6-dien-26-oic acid^6^ | 15.11 | 7.41 | 19.02 | 7.55 |
| Cholest-5-ene-3β,24S-diol^3^ (24S-hydroxycholesterol) | 13.01 | 4.41 | 10.70 | 3.29 |
| 3β,22,25-Trihydroxycholest-5-en-24-one^1^ | 6.60 | 1.94 | 6.80 | 3.67 |
| 3β,7β-Dihydroxycholest-5-en-26-oic acid^3^ | 4.48 | 2.35 | 4.71 | 0.99 |
| 7α-Hydroxy-3-oxochol-4-en-24-oic acid | 4.35 | 2.12 | 4.55 | 1.73 |
| 3β-Hydroxychol-5-en-24-oic acid | 3.24 | 1.09 | 3.04 | 1.02 |
| 3β,7α-Dihydroxychol-5-en-24-oic acid | 3.11 | 2.14 | 3.17 | 1.94 |
| 7α,26-Dihydroxycholest-4-en-3-one | 2.43 | 0.98 | 2.46 | 0.79 |
| Cholest-5-ene-3β,25-diol^3^ (25-hydroxycholesterol) | 1.97 | 0.38 | 2.19 | 1.29 |
| 3-Oxocholest-4-en-26-oic acid | 1.78 | 0.53 | 2.51 | 0.91 |
| 3β-Hydroxycholesta-5,7-dien-26-oic acid^7^ | 1.12 | 1.53 | 1.81 | 1.55 |
| 7α,25-Dihydroxycholest-4-en-3-one | 0.79 | 0.29 | 1.10 | 0.40 |
| 7α-Hydroxy-26-nor-cholest-4-ene-3,24-dione^1,2^ | 0.35 | 0.12 | 0.38 | 0.11 |
| Cholest-5-ene-3β,7α,26-triol (7α,26-Dihydroxycholesterol) | 0.31 | 0.26 | 0.27 | 0.24 |
| Cholest-5-ene-3β,7α,25-triol (7α,25-Dihydroxycholesterol) | 0.21 | 0.13 | 0.24 | 0.14 |

**Table E6­­­­­­­­. Oxysterols and cholestenoic acids in human IPF and control sera.** Oxysterols and cholestenoic acids identified by LC-ESI-MS^n^ in serum following solid phase extraction and charge-tagging with GP-hydrazine. In the absence of authentic standards presumptive identifications based on exact mass, MS^n^ spectra and retention time are given. Quantitation was by stable isotope dilution.

We have adopted the systematic sterol nomenclature recommended by the Lipid Maps consortium http://www.lipidmaps.org/. In this nomenclature hydroxylation of the terminal carbon of the sterol side-chain introducing R stereochemistry at C-25 is defined as C-26 hydroxylation.

^1^ Presumptive identification based on exact mass and MS^n^ spectra. ^2^ 26-Nor-sterol is a likely decomposition product of a 24-oxo-26 acid. ^3^ LXR ligand. ^4^ May be formed by autoxidation. ^5^ Can be formed enzymatically and by autoxidation. ^6^ 7α-Hydroxy-3-oxocholest-4-en-26-oic acid dehydrates to a minor degree to 3-oxocholesta-4,6-dien-26-oic acid. Thus, the total 7α-hydroxy-3-oxocholest-4-en-26-oic acid corresponds to the sum of the two acids. ^7^ 3β,7α-Dihydroxycholest-5-en-26-oic acid dehydrates to a minor degree to 3β-hydroxycholesta-5,7-dien-26-oic acid. Thus, the total 3β,7α-dihydroxycholest-5-en-26-oic acid corresponds to the sum of the two acids. To be noted 27-hydroxycholesterol is (25R)26-hydroxycholesterol according to IUPAC nomenclature.

|  | **Forward** 5'--3' | **Reverse** 5'--3'/Company |
| --- | --- | --- |
| **Mouse** |  |  |
| TBP | TGC TGT TGG TGA TTG TTG GT | AAC TGG CTT GTG TGG GAA AG |
| 18S | 4310893E | Life Technologies |
| Col1A1 | AGC TTT GTG GAC CTC CGG CT | ACA CAG CCG TGC CAT TGT GG |
| Col3A1 | GTT CTA GAG GAT GGC TGT ACT A AA CAC A | TTG CCT TGC GTG TTT GAT ATT C |
| Col1A1 | Mm00801666_m1 | Life Technologies |
| Col1A2 | Mm00483888_m1 | Life Technologies |
| Tgfβ1 | ACC CCC CAT TGCT GTC CCGT | CCT TGG TTC AGC CAC TGC CG |
| Abca1 | Mm00442646_m1 | Life Technologies |
| Rxrα1 | Mm00441185_m1 | Life Technologies |
| Arg2 | ACC AGG AAC TGG CTG AAG TG | TGA GCA TCA ACC CAG ATG AC |
| Lxrβ (NR1H2) | Mm00443451_m1 | Life Technologies |
| Lxrα (NR1H3) | GCT CAG GAG CTG ATG ATC CA | GCG CTT GAT CCT CGT GTA G |
| Il13rα2 | TCT GGT ATG AGG GCT TGG AT | GCT GGA GGT AAT CAG CAC ACT |
| Ym1 | CAT GAG CAA GAC TTG CGT GAC | GGT CCA AAC TTC CAT CCT CCA |
| Nos2 | AGA CCT CAA CAG AGC CCT CA | GCA GCC TCT TGT CTT TGA CC |
| miR-155 | TM002623/MS00031486 | Life Technologies/Qiagen |
| RNU6 | TM001973/MS00033740 | Life Technologies/Qiagen |
| Hif1α | Mm00468869_m1 | Life Technologies |
| **Human** |  |  |
| β-actin | 4310881E | Life Technologies |
| COL1A1 | CAA TGC TGC CCT TTC TGC TCC TTT | CAC TTG GGT GTT TGA GCA TTG CCT |
| COL3A1 | TAT CGA ACA CGC AAG GCT GTG AGA | GGC CAA CGT CCA CAC CAA ATT CTT |
| LXRα (NR1H3) | Hs00172885_m1 | Life Technologies |
| ABCA1 | Hs01059118_m1 | Life Technologies |
| ARG2 | Hs00982833_m1 | Life Technologies |
| miR-155 | TM002623/MS00031486 | Life Technologies/Qiagen |
| ZNF652 | Hs00977533_m1 | Life Technologies |

**Table E7. Primer sequences for TaqMan assays**

References

1. Kurowska-Stolarska M, Alivernini S, Ballantine LE, Asquith DL, Millar NL, Gilchrist DS, et al. MicroRNA-155 as a proinflammatory regulator in clinical and experimental arthritis. Proc Natl Acad Sci U S A 2011; 108:11193-8.

2. Tranheim Kase E, Nikolic N, Pettersen Hessvik N, Fjeldheim AK, Jensen J, Thoresen GH, et al. Dietary supplementation with 22-S-hydroxycholesterol to rats reduces body weight gain and the accumulation of liver triacylglycerol. Lipids 2012; 47:483-93.

3. Moeller A, Ask K, Warburton D, Gauldie J, Kolb M. The bleomycin animal model: a useful tool to investigate treatment options for idiopathic pulmonary fibrosis? The international journal of biochemistry & cell biology 2008; 40:362-82.

4. B BM, Lawson WE, Oury TD, Sisson TH, Raghavendran K, Hogaboam CM. Animal models of fibrotic lung disease. American journal of respiratory cell and molecular biology 2013; 49:167-79.

5. Li M, Krishnaveni MS, Li C, Zhou B, Xing Y, Banfalvi A, et al. Epithelium-specific deletion of TGF-beta receptor type II protects mice from bleomycin-induced pulmonary fibrosis. The Journal of clinical investigation 2011; 121:277-87.

6. Kurowska-Stolarska M, Stolarski B, Kewin P, Murphy G, Corrigan CJ, Ying S, et al. IL-33 amplifies the polarization of alternatively activated macrophages that contribute to airway inflammation. J Immunol 2009; 183:6469-77.

7. Patel M, Xu D, Kewin P, Choo-Kang B, McSharry C, Thomson NC, et al. TLR2 agonist ameliorates established allergic airway inflammation by promoting Th1 response and not via regulatory T cells. Journal of immunology 2005; 174:7558-63.

8. Hsu E, Shi H, Jordan RM, Lyons-Weiler J, Pilewski JM, Feghali-Bostwick CA. Lung tissues in patients with systemic sclerosis have gene expression patterns unique to pulmonary fibrosis and pulmonary hypertension. Arthritis and rheumatism 2011; 63:783-94.

9. Pilewski JM, Liu L, Henry AC, Knauer AV, Feghali-Bostwick CA. Insulin-like growth factor binding proteins 3 and 5 are overexpressed in idiopathic pulmonary fibrosis and contribute to extracellular matrix deposition. The American journal of pathology 2005; 166:399-407.

10. Welsh DJ, Peacock AJ, MacLean M, Harnett M. Chronic hypoxia induces constitutive p38 mitogen-activated protein kinase activity that correlates with enhanced cellular proliferation in fibroblasts from rat pulmonary but not systemic arteries. American journal of respiratory and critical care medicine 2001; 164:282-9.

11. Malhotra D, Fletcher AL, Astarita J, Lukacs-Kornek V, Tayalia P, Gonzalez SF, et al. Transcriptional profiling of stroma from inflamed and resting lymph nodes defines immunological hallmarks. Nat Immunol 2012; 13:499-510.

12. Costello CM, Howell K, Cahill E, McBryan J, Konigshoff M, Eickelberg O, et al. Lung-selective gene responses to alveolar hypoxia: potential role for the bone morphogenetic antagonist gremlin in pulmonary hypertension. American journal of physiology. Lung cellular and molecular physiology 2008; 295:L272-84.

13. Yoon JH, Srikantan S, Gorospe M. MS2-TRAP (MS2-tagged RNA affinity purification): tagging RNA to identify associated miRNAs. Methods 2012; 58:81-7.

14. Griffiths WJ, Crick PJ, Wang Y, Ogundare M, Tuschl K, Morris AA, et al. Analytical strategies for characterization of oxysterol lipidomes: liver X receptor ligands in plasma. Free radical biology & medicine 2013; 59:69-84.

15. Martin MM, Lee EJ, Buckenberger JA, Schmittgen TD, Elton TS. MicroRNA-155 regulates human angiotensin II type 1 receptor expression in fibroblasts. The Journal of biological chemistry 2006; 281:18277-84.

16. Rai D, Kim SW, McKeller MR, Dahia PL, Aguiar RC. Targeting of SMAD5 links microRNA-155 to the TGF-beta pathway and lymphomagenesis. Proc Natl Acad Sci U S A; 107:3111-6.

17. Louafi F, Martinez-Nunez RT, Sanchez-Elsner T. MicroRNA-155 targets SMAD2 and modulates the response of macrophages to transforming growth factor-{beta}. The Journal of biological chemistry 2010; 285:41328-36.

18. Bruning U, Cerone L, Neufeld Z, Fitzpatrick SF, Cheong A, Scholz CC, et al. MicroRNA-155 promotes resolution of hypoxia-inducible factor 1alpha activity during prolonged hypoxia. Molecular and cellular biology 2011; 31:4087-96.
