# Supplementary material for: The role of microRNA-155/liver X receptor pathway in experimental and idiopathic pulmonary fibrosis
Source: J Allergy Clin Immunol. 2017 Jun;139(6):1946–56. doi: 10.1016/j.jaci.2016.09.021 (PMC5457127; doi:10.1016/j.jaci.2016.09.021)
Supplement: Legends for Figures E1-E9 [file mmc2.docx]

**Mariola Kurowska-Stolarska et al**

**The role of microRNA-155/LXR pathway in experimental and Idiopathic Pulmonary Fibrosis**

**Supplementary Figure Legends**

**FIG E1**. miR-155^-/-^  bleo mice show an increase in the expression of lung collagen 1a *(Col1a1)* but not collagen1a2 (*Col1a2*). Wild-type and miR-155^-/-^ mice were given bleomycin (bleo) or PBS control (n=8 per group) on day 1 and tissues harvested on day 18. QPCR data were normalised to endogenous control (18S); and presented as 2^-deltadeltaCT^ (fold change relative to mean of WT PBS group). Data are shown as median and inter-quartile range from 2 independent experiments. *P<0.05.

**FIG E2.** Regulation of miR-155 in murine lung fibroblasts. (**A to C)** Primary fibroblasts from pooled lungs of WT mice (n=5) were synchronized in culture with serum-free medium and then cultured with bleomycin (1 and 10 μg/ml), or IL-33, IL-1α, IL-25 or HMGB1 (10 or 100 ng/ml) for 8 and 24h. Cells were collected and expression of miR-155 evaluated by qPCR. **(A)** Bleomycin inhibited the expression of miR-155 at 24h, **(B)** Exogenous IL-33, IL-25 and HMGB-1 did not affect miR-155 expression. **(C)** Exogenous IL-1α increased miR-155 expression. Data presented as mean ± SEM of 3 technical replicates, repeated in 2 independent experiments. *P<0.05.

**FIG E3.** Lung tissue *Hif1α*, *TGFβ* and *Smad1* mRNA expression was increased in miR-155^-/-^ mice given bleomycin. RNA was purified from lung tissue harvested on day 18 from wild-type (WT) and miR-155^-/-^ mice given bleomycin or control PBS (n=8 per group). The expression levels of *Hif1α, Tgfβ* and *Smad1* were evaluated by qPCR and presented as relative to 18S (2^-deltaCT^). All genes tested were up-regulated in bleomycin treated miR-155^-/-^ compared to WT. Data are presented as box and whisker plots showing median and inter-quartile range. * P<0.05.

**FIG E4.** LXRα protein expression in lung cells during bleomycin induced fibrosis. **(A-D)** WT mice were given bleomycin (n=24) or PBS (n=7) on day 0 and lungs were harvested on days: 1, 2 ,3, 7 and 10. Lungs from 4-5 mice (bleo group) and 1-2 mice (PBS group) were harvested at each time point; and LXRα expression evaluated by flow cytometry. Expression of LXRα in PBS group remained stable through the time course of experiment. **(A)** Gating strategy for macrophages, fibroblasts and epithelial cells in digested lungs. **(B)** Representative and quantitative expression of LXRα in small macrophage population (Mac-1) showing no difference between PBS and bleo group at any time points. **(C)** Representative and quantitative expression of LXRα in large macrophage population (Mac-2) showing no difference between PBS and bleo group at any time points. (D) Representative and quantitative expression of LXRα in epithelial cells showing no difference between PBS and bleo group at any time points. Data are presented as % of median fluorescence intensity (MFI) of LXRα in PBS groups after subtraction of isotype MFI ± SEM of biological replicates *P<0.05.

**FIG E5**. Validation of transfection efficiency of *LXR* siRNA. **(A)** Wild-type and miR-155^-/-^ lung macrophages (n=3), **(B)** lung fibroblasts (pooled n=4), **(C)** human blood monocyte-derived macrophages (n=2) prior transfected with control inhibitor (Ci) or miR-155 inhibitor (miR155i) and **(D)** human IPF primary lung fibroblasts (n=3) were transfected with control siRNA or siRNA for *LXR* (both 50 nM). All data were collected 48 h after transfection. Expression of *LXRa* quantified by qPCR are normalised to 18S (mouse) or *β−actin* (human). Data are presented as change in *LXRa* expression compared to cells transfected with control (C) siRNA.

**FIG E6**. Inhibition of miR-155 in human macrophages by gene-silencing leads to increased LXR-dependent *ARG2* mRNA expression. Macrophages were cultured (as in Fig E5, C) for 16h after siRNA transfection. GW3965 (4μM) or excipient control DMSO was then added for a further 24h. Inhibition of miR-155 in human macrophages enhanced LXR agonist-induced *ARG2* expression. Values quantified by qPCR were normalized to *β-actin* and presented as 2^-deltaCT^. Histograms show mean ± SEM of three technical replicates. *P<0.05.

**FIG E7**. The bleomycin-induced bronchoalveolar lavage leukocytosis in miR-155^-/-^ mice was reduced by 22(S) hydroxycholesterol treatment. Airway and alveolar leukocytes were harvested by bronchoalveolar lavage (BAL) from WT and miR-155^-/-^ mice on day 18 after bleomycin or PBS (as in Fig 4, A). The increased BAL leukocytosis in miR-155^-/-^ mice given bleomycin was reduced when treated with the LXR antagonist 22(S) hydroxy-cholesterol [22(S)HC] compared with control excipient (40% 2-hydroxypropyl-β-cyclodextrin in water), and was then not different from wild-type mice given bleomycin and treated with excipient. Histograms show mean ± SEM of 6-10 mice. *P<0.05.

**FIG E8**. LXR antagonist reduces IPF fibroblast proliferation and migration. **(A to C)** Normal and IPF primary lung fibroblasts were serum-starved for 24h to synchronize their growth and then medium containing 1% FCS with or without 22(S)HC was added for 48h. **(A)** Cells were pulsed with (^3^H) thymidine 4 hours before harvest. IPF fibroblasts had higher than normal proliferation and this was reduced to normal by 22(S)HC. **(B to C)** Fibroblast migration measured in a wound-healing assay. A uniform scratch was made through confluent layers of fibroblasts that were then cultured with zero-percent FCS or 0.3% FCS with 22(H)HC or excipient DMSO and after 24h the breadth of the scratch space was measured. **(B)** A typical image showing that IPF fibroblasts demonstrate higher migration than normal fibroblasts into the scratch space. **(C)** Migration was quantified and presented as mean ± SEM of 2-3 biological replicates. Each sample was done in technical replicates (n=4). *P<0.05.

**FIG E9.** Coordinated expression of miR-155 and LXRα is deregulated by hypoxia in IPF fibroblasts. **(A)** Synchronized normal (n=3) and IPF (n=3) fibroblasts were transfected with control miR or miR-155 (C = control miR mimic; miR-155 = miR-155 mimic) and cultured in 1% FCS for 16h then supplemented with GW3965 for 24h and collagen measured by colorimetric (Sircol) assay. **(B)** Primary lung fibroblast cell lines from control donors (n=8) and IPF (n=7) were expanded to passage #4, then synchronized in serum-free medium and cultured in medium containing 1% FSC (%S) for 48h. The expression of miR-155 was quantified by qPCR. **(C-D)** Control (n=4) and IPF (n=4) lung fibroblast cultures were synchronized in serum-free medium for 24h which was then replaced with fresh serum free media or medium containing 1% FSC for a further 48h in 21% oxygen; normoxia (N) or 1% oxygen; hypoxia (H). The expression of **(C)** miR-155 and **(D)** mRNA for *LXRa* and *ABCA1* were quantified by qPCR. **(E)** The individual expression levels for miR-155 and *LXRα* from normal (n=4) and IPF (n=4) fibroblasts cultured in serum free media or media containing 1% FCS in two separated experiments under hypoxia were plotted as dot-plot graph. **(F)** Cells were cultured as in C and *ZNF652* expression evaluated by qPCR. **(G)** The individual expression levels for miR-155 and *ZNF652* from normal and IPF fibroblasts cultured under hypoxia were plotted as dot-plot graph. Values quantified by qPCR relative to *β-actin* (mRNA) or snU6 (miR-155). Data are presented as dot plots with a mean bar, or mean ± SEM. *P<0.05; each individual was done in technical (n=2) and experimental (n=2) replicates; solid circles represent fibroblasts in medium containing 1% FSC; solid triangles represent fibroblasts, in serum-free medium.
