# Supplementary figures and images for: The role of microRNA-155/liver X receptor pathway in experimental and idiopathic pulmonary fibrosis
Source: J Allergy Clin Immunol. 2017 Jun;139(6):1946–56. doi: 10.1016/j.jaci.2016.09.021 (PMC5457127; doi:10.1016/j.jaci.2016.09.021)

Figure E1

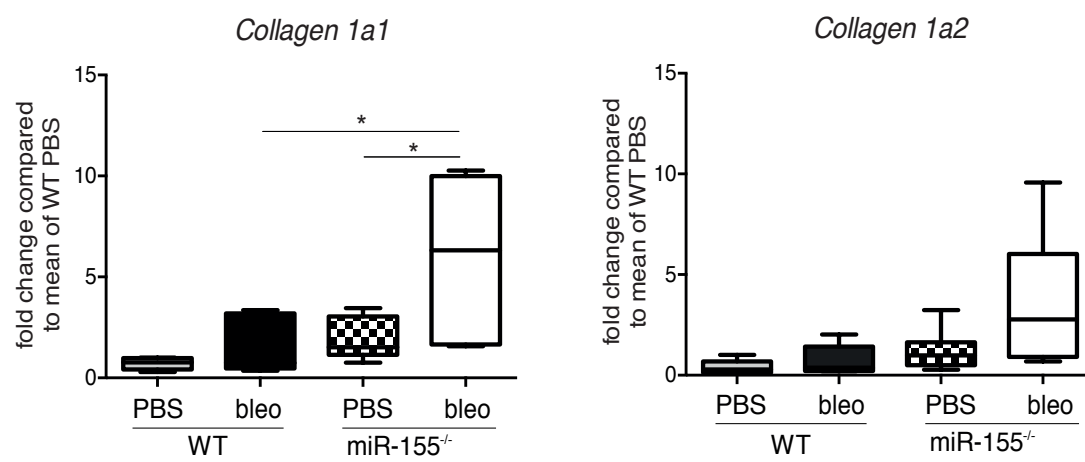

Supplement: Fig E1 [file mmc3.pdf]

Figure E2

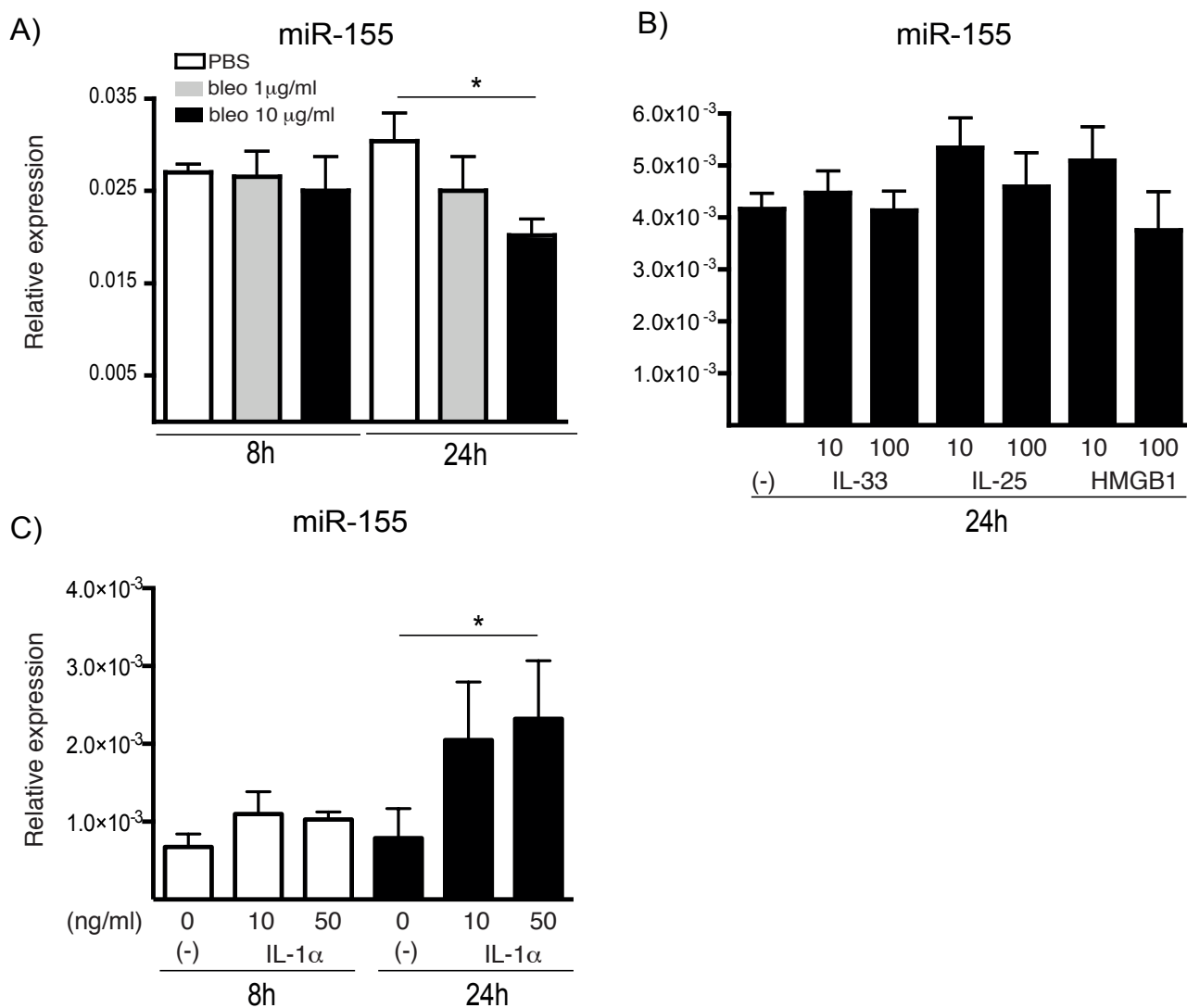

Supplement: Fig E2 [file mmc4.pdf]

Figure E3

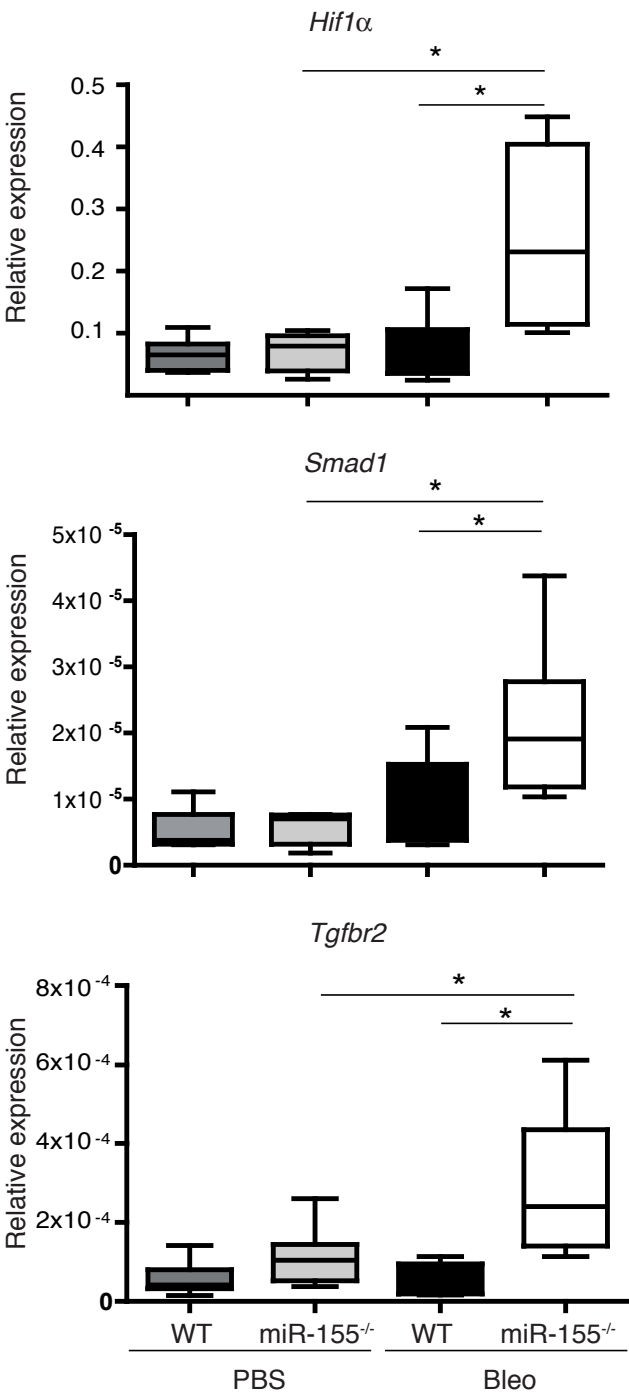

Supplement: Fig E3 [file mmc5.pdf]

Figure E4

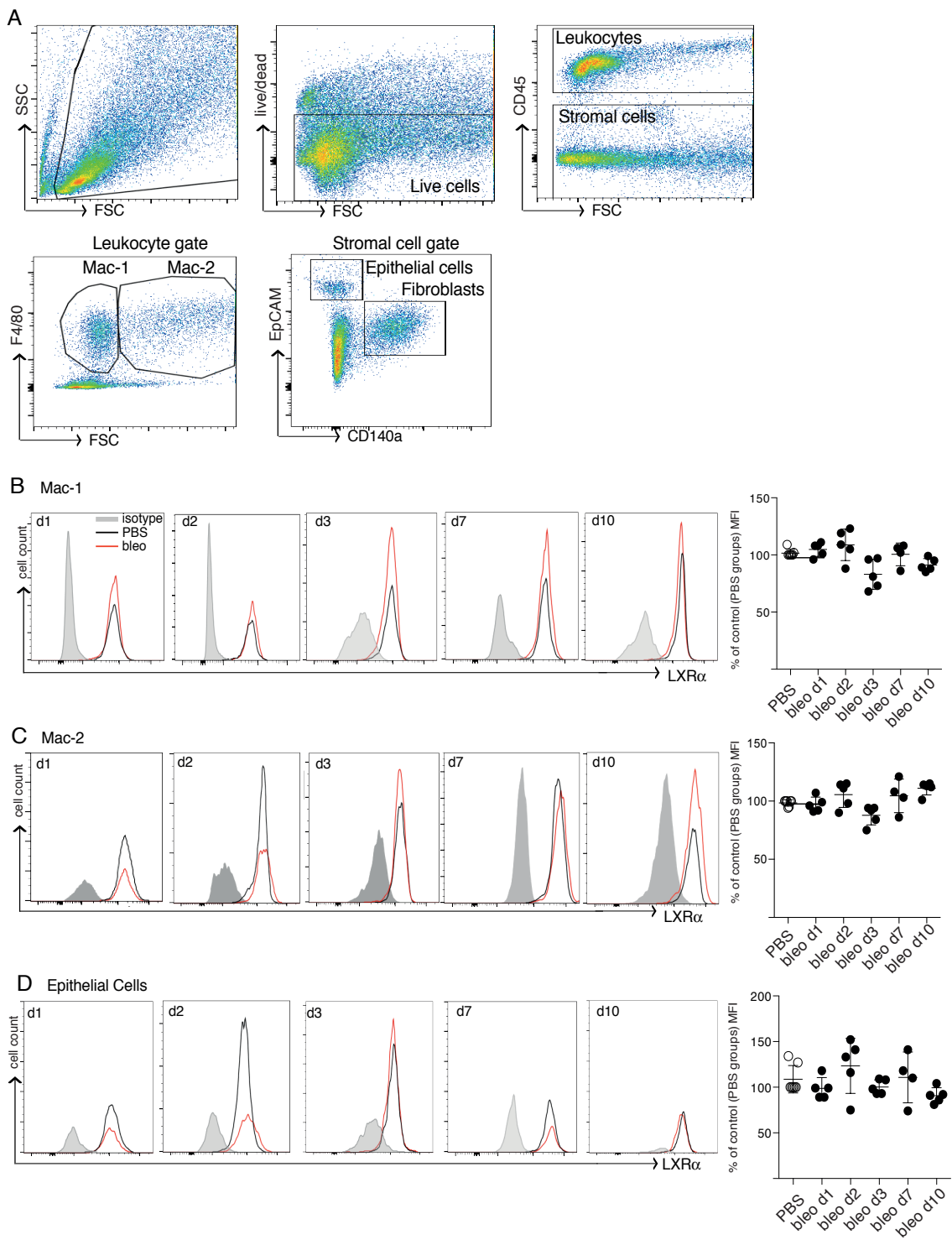

Supplement: Fig E4 [file mmc6.pdf]

Figure E5

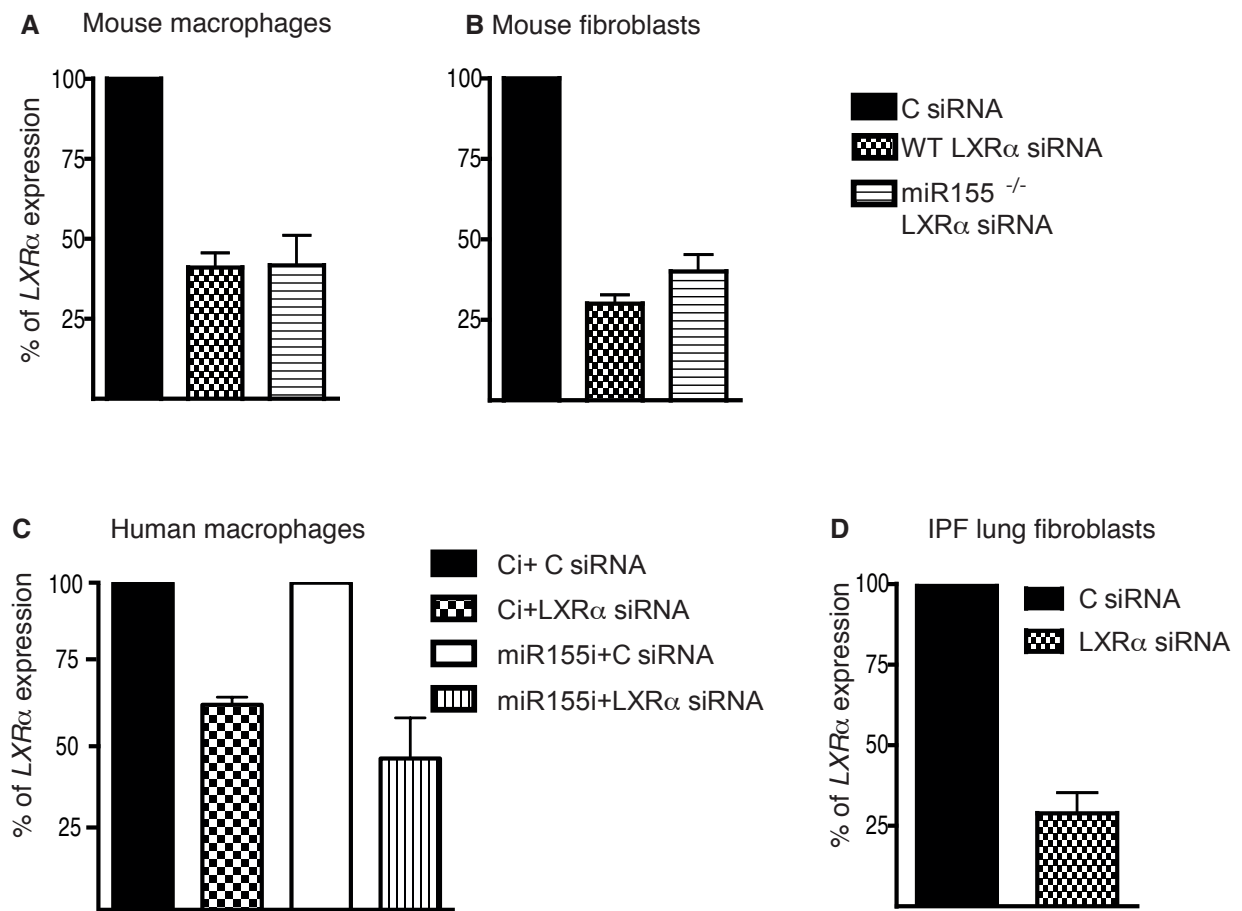

Supplement: Fig E5 [file mmc7.pdf]

Figure E6

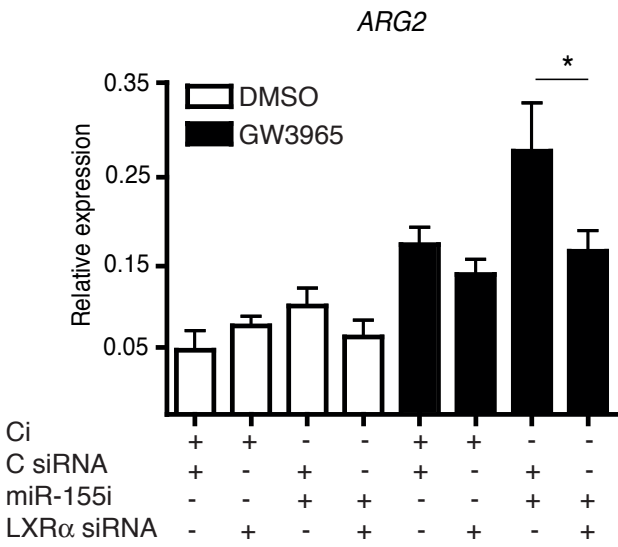

Supplement: Fig E6 [file mmc8.pdf]

Figure E7

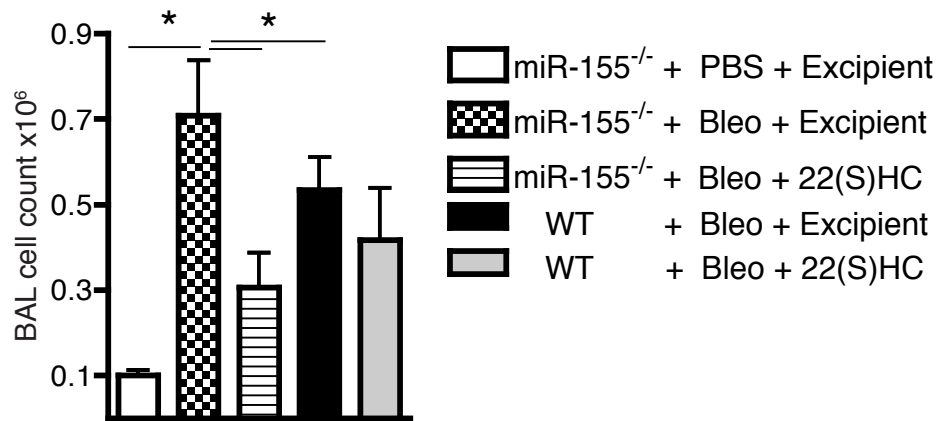

Supplement: Fig E7 [file mmc9.pdf]

Figure E8

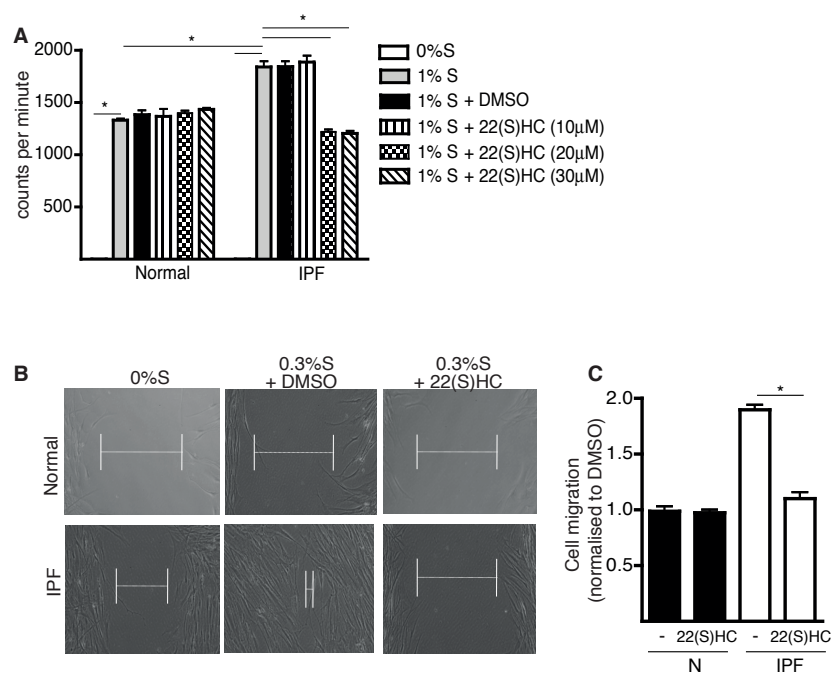

Supplement: Fig E8 [file mmc10.pdf]

Figure E9

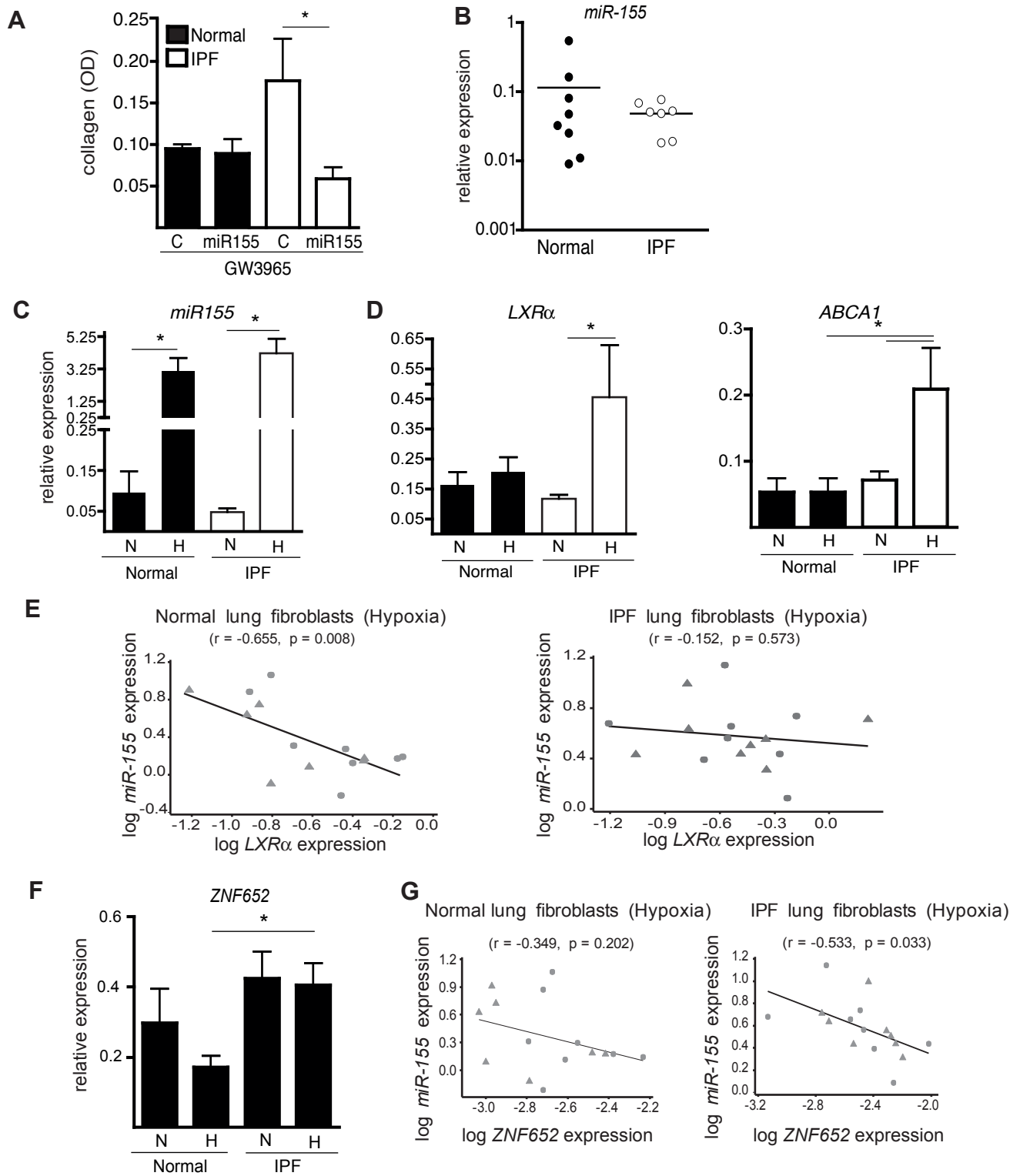

Supplement: Fig E9 [file mmc11.pdf]
